# Search for triboson $W^{\pm }W^{\pm }W^{\mp }$ production in *pp* collisions at $\sqrt{s}=8$ $\text {TeV}$ with the ATLAS detector

**DOI:** 10.1140/epjc/s10052-017-4692-1

**Published:** 2017-03-02

**Authors:** M. Aaboud, G. Aad, B. Abbott, J. Abdallah, O. Abdinov, B. Abeloos, R. Aben, O. S. AbouZeid, N. L. Abraham, H. Abramowicz, H. Abreu, R. Abreu, Y. Abulaiti, B. S. Acharya, S. Adachi, L. Adamczyk, D. L. Adams, J. Adelman, S. Adomeit, T. Adye, A. A. Affolder, T. Agatonovic-Jovin, J. Agricola, J. A. Aguilar-Saavedra, S. P. Ahlen, F. Ahmadov, G. Aielli, H. Akerstedt, T. P. A. Åkesson, A. V. Akimov, G. L. Alberghi, J. Albert, S. Albrand, M. J. Alconada Verzini, M. Aleksa, I. N. Aleksandrov, C. Alexa, G. Alexander, T. Alexopoulos, M. Alhroob, B. Ali, M. Aliev, G. Alimonti, J. Alison, S. P. Alkire, B. M. M. Allbrooke, B. W. Allen, P. P. Allport, A. Aloisio, A. Alonso, F. Alonso, C. Alpigiani, A. A. Alshehri, M. Alstaty, B. Alvarez Gonzalez, D. Álvarez Piqueras, M. G. Alviggi, B. T. Amadio, K. Amako, Y. Amaral Coutinho, C. Amelung, D. Amidei, S. P. Amor Dos Santos, A. Amorim, S. Amoroso, G. Amundsen, C. Anastopoulos, L. S. Ancu, N. Andari, T. Andeen, C. F. Anders, G. Anders, J. K. Anders, K. J. Anderson, A. Andreazza, V. Andrei, S. Angelidakis, I. Angelozzi, P. Anger, A. Angerami, F. Anghinolfi, A. V. Anisenkov, N. Anjos, A. Annovi, C. Antel, M. Antonelli, A. Antonov, F. Anulli, M. Aoki, L. Aperio Bella, G. Arabidze, Y. Arai, J. P. Araque, A. T. H. Arce, F. A. Arduh, J-F. Arguin, S. Argyropoulos, M. Arik, A. J. Armbruster, L. J. Armitage, O. Arnaez, H. Arnold, M. Arratia, O. Arslan, A. Artamonov, G. Artoni, S. Artz, S. Asai, N. Asbah, A. Ashkenazi, B. Åsman, L. Asquith, K. Assamagan, R. Astalos, M. Atkinson, N. B. Atlay, K. Augsten, G. Avolio, B. Axen, M. K. Ayoub, G. Azuelos, M. A. Baak, A. E. Baas, M. J. Baca, H. Bachacou, K. Bachas, M. Backes, M. Backhaus, P. Bagiacchi, P. Bagnaia, Y. Bai, J. T. Baines, O. K. Baker, E. M. Baldin, P. Balek, T. Balestri, F. Balli, W. K. Balunas, E. Banas, Sw. Banerjee, A. A. E. Bannoura, L. Barak, E. L. Barberio, D. Barberis, M. Barbero, T. Barillari, M-S Barisits, T. Barklow, N. Barlow, S. L. Barnes, B. M. Barnett, R. M. Barnett, Z. Barnovska-Blenessy, A. Baroncelli, G. Barone, A. J. Barr, L. Barranco Navarro, F. Barreiro, J. Barreiro Guimarães da Costa, R. Bartoldus, A. E. Barton, P. Bartos, A. Basalaev, A. Bassalat, R. L. Bates, S. J. Batista, J. R. Batley, M. Battaglia, M. Bauce, F. Bauer, H. S. Bawa, J. B. Beacham, M. D. Beattie, T. Beau, P. H. Beauchemin, P. Bechtle, H. P. Beck, K. Becker, M. Becker, M. Beckingham, C. Becot, A. J. Beddall, A. Beddall, V. A. Bednyakov, M. Bedognetti, C. P. Bee, L. J. Beemster, T. A. Beermann, M. Begel, J. K. Behr, C. Belanger-Champagne, A. S. Bell, G. Bella, L. Bellagamba, A. Bellerive, M. Bellomo, K. Belotskiy, O. Beltramello, N. L. Belyaev, O. Benary, D. Benchekroun, M. Bender, K. Bendtz, N. Benekos, Y. Benhammou, E. Benhar Noccioli, J. Benitez, D. P. Benjamin, J. R. Bensinger, S. Bentvelsen, L. Beresford, M. Beretta, D. Berge, E. Bergeaas Kuutmann, N. Berger, J. Beringer, S. Berlendis, N. R. Bernard, C. Bernius, F. U. Bernlochner, T. Berry, P. Berta, C. Bertella, G. Bertoli, F. Bertolucci, I. A. Bertram, C. Bertsche, D. Bertsche, G. J. Besjes, O. Bessidskaia Bylund, M. Bessner, N. Besson, C. Betancourt, A. Bethani, S. Bethke, A. J. Bevan, R. M. Bianchi, L. Bianchini, M. Bianco, O. Biebel, D. Biedermann, R. Bielski, N. V. Biesuz, M. Biglietti, J. Bilbao De Mendizabal, T. R. V. Billoud, H. Bilokon, M. Bindi, S. Binet, A. Bingul, C. Bini, S. Biondi, T. Bisanz, D. M. Bjergaard, C. W. Black, J. E. Black, K. M. Black, D. Blackburn, R. E. Blair, J.-B. Blanchard, T. Blazek, I. Bloch, C. Blocker, A. Blue, W. Blum, U. Blumenschein, S. Blunier, G. J. Bobbink, V. S. Bobrovnikov, S. S. Bocchetta, A. Bocci, C. Bock, M. Boehler, D. Boerner, J. A. Bogaerts, D. Bogavac, A. G. Bogdanchikov, C. Bohm, V. Boisvert, P. Bokan, T. Bold, A. S. Boldyrev, M. Bomben, M. Bona, M. Boonekamp, A. Borisov, G. Borissov, J. Bortfeldt, D. Bortoletto, V. Bortolotto, K. Bos, D. Boscherini, M. Bosman, J. D. Bossio Sola, J. Boudreau, J. Bouffard, E. V. Bouhova-Thacker, D. Boumediene, C. Bourdarios, S. K. Boutle, A. Boveia, J. Boyd, I. R. Boyko, J. Bracinik, A. Brandt, G. Brandt, O. Brandt, U. Bratzler, B. Brau, J. E. Brau, W. D. Breaden Madden, K. Brendlinger, A. J. Brennan, L. Brenner, R. Brenner, S. Bressler, T. M. Bristow, D. Britton, D. Britzger, F. M. Brochu, I. Brock, R. Brock, G. Brooijmans, T. Brooks, W. K. Brooks, J. Brosamer, E. Brost, J. H Broughton, P. A. Bruckman de Renstrom, D. Bruncko, R. Bruneliere, A. Bruni, G. Bruni, L. S. Bruni, B. H. Brunt, M. Bruschi, N. Bruscino, P. Bryant, L. Bryngemark, T. Buanes, Q. Buat, P. Buchholz, A. G. Buckley, I. A. Budagov, F. Buehrer, M. K. Bugge, O. Bulekov, D. Bullock, H. Burckhart, S. Burdin, C. D. Burgard, B. Burghgrave, K. Burka, S. Burke, I. Burmeister, J. T. P. Burr, E. Busato, D. Büscher, V. Büscher, P. Bussey, J. M. Butler, C. M. Buttar, J. M. Butterworth, P. Butti, W. Buttinger, A. Buzatu, A. R. Buzykaev, S. Cabrera Urbán, D. Caforio, V. M. Cairo, O. Cakir, N. Calace, P. Calafiura, A. Calandri, G. Calderini, P. Calfayan, G. Callea, L. P. Caloba, S. Calvente Lopez, D. Calvet, S. Calvet, T. P. Calvet, R. Camacho Toro, S. Camarda, P. Camarri, D. Cameron, R. Caminal Armadans, C. Camincher, S. Campana, M. Campanelli, A. Camplani, A. Campoverde, V. Canale, A. Canepa, M. Cano Bret, J. Cantero, T. Cao, M. D. M. Capeans Garrido, I. Caprini, M. Caprini, M. Capua, R. M. Carbone, R. Cardarelli, F. Cardillo, I. Carli, T. Carli, G. Carlino, L. Carminati, S. Caron, E. Carquin, G. D. Carrillo-Montoya, J. R. Carter, J. Carvalho, D. Casadei, M. P. Casado, M. Casolino, D. W. Casper, E. Castaneda-Miranda, R. Castelijn, A. Castelli, V. Castillo Gimenez, N. F. Castro, A. Catinaccio, J. R. Catmore, A. Cattai, J. Caudron, V. Cavaliere, E. Cavallaro, D. Cavalli, M. Cavalli-Sforza, V. Cavasinni, F. Ceradini, L. Cerda Alberich, B. C. Cerio, A. S. Cerqueira, A. Cerri, L. Cerrito, F. Cerutti, M. Cerv, A. Cervelli, S. A. Cetin, A. Chafaq, D. Chakraborty, S. K. Chan, Y. L. Chan, P. Chang, J. D. Chapman, D. G. Charlton, A. Chatterjee, C. C. Chau, C. A. Chavez Barajas, S. Che, S. Cheatham, A. Chegwidden, S. Chekanov, S. V. Chekulaev, G. A. Chelkov, M. A. Chelstowska, C. Chen, H. Chen, K. Chen, S. Chen, S. Chen, X. Chen, Y. Chen, H. C. Cheng, H. J Cheng, Y. Cheng, A. Cheplakov, E. Cheremushkina, R. Cherkaoui El Moursli, V. Chernyatin, E. Cheu, L. Chevalier, V. Chiarella, G. Chiarelli, G. Chiodini, A. S. Chisholm, A. Chitan, M. V. Chizhov, K. Choi, A. R. Chomont, S. Chouridou, B. K. B. Chow, V. Christodoulou, D. Chromek-Burckhart, J. Chudoba, A. J. Chuinard, J. J. Chwastowski, L. Chytka, G. Ciapetti, A. K. Ciftci, D. Cinca, V. Cindro, I. A. Cioara, C. Ciocca, A. Ciocio, F. Cirotto, Z. H. Citron, M. Citterio, M. Ciubancan, A. Clark, B. L. Clark, M. R. Clark, P. J. Clark, R. N. Clarke, C. Clement, Y. Coadou, M. Cobal, A. Coccaro, J. Cochran, L. Colasurdo, B. Cole, A. P. Colijn, J. Collot, T. Colombo, G. Compostella, P. Conde Muiño, E. Coniavitis, S. H. Connell, I. A. Connelly, V. Consorti, S. Constantinescu, G. Conti, F. Conventi, M. Cooke, B. D. Cooper, A. M. Cooper-Sarkar, K. J. R. Cormier, T. Cornelissen, M. Corradi, F. Corriveau, A. Cortes-Gonzalez, G. Cortiana, G. Costa, M. J. Costa, D. Costanzo, G. Cottin, G. Cowan, B. E. Cox, K. Cranmer, S. J. Crawley, G. Cree, S. Crépé-Renaudin, F. Crescioli, W. A. Cribbs, M. Crispin Ortuzar, M. Cristinziani, V. Croft, G. Crosetti, A. Cueto, T. Cuhadar Donszelmann, J. Cummings, M. Curatolo, J. Cúth, H. Czirr, P. Czodrowski, G. D’amen, S. D’Auria, M. D’Onofrio, M. J. Da Cunha Sargedas De Sousa, C. Da Via, W. Dabrowski, T. Dado, T. Dai, O. Dale, F. Dallaire, C. Dallapiccola, M. Dam, J. R. Dandoy, N. P. Dang, A. C. Daniells, N. S. Dann, M. Danninger, M. Dano Hoffmann, V. Dao, G. Darbo, S. Darmora, J. Dassoulas, A. Dattagupta, W. Davey, C. David, T. Davidek, M. Davies, P. Davison, E. Dawe, I. Dawson, K. De, R. de Asmundis, A. De Benedetti, S. De Castro, S. De Cecco, N. De Groot, P. de Jong, H. De la Torre, F. De Lorenzi, A. De Maria, D. De Pedis, A. De Salvo, U. De Sanctis, A. De Santo, J. B. De Vivie De Regie, W. J. Dearnaley, R. Debbe, C. Debenedetti, D. V. Dedovich, N. Dehghanian, I. Deigaard, M. Del Gaudio, J. Del Peso, T. Del Prete, D. Delgove, F. Deliot, C. M. Delitzsch, A. Dell’Acqua, L. Dell’Asta, M. Dell’Orso, M. Della Pietra, D. della Volpe, M. Delmastro, P. A. Delsart, D. A. DeMarco, S. Demers, M. Demichev, A. Demilly, S. P. Denisov, D. Denysiuk, D. Derendarz, J. E. Derkaoui, F. Derue, P. Dervan, K. Desch, C. Deterre, K. Dette, P. O. Deviveiros, A. Dewhurst, S. Dhaliwal, A. Di Ciaccio, L. Di Ciaccio, W. K. Di Clemente, C. Di Donato, A. Di Girolamo, B. Di Girolamo, B. Di Micco, R. Di Nardo, A. Di Simone, R. Di Sipio, D. Di Valentino, C. Diaconu, M. Diamond, F. A. Dias, M. A. Diaz, E. B. Diehl, J. Dietrich, S. Díez Cornell, A. Dimitrievska, J. Dingfelder, P. Dita, S. Dita, F. Dittus, F. Djama, T. Djobava, J. I. Djuvsland, M. A. B. do Vale, D. Dobos, M. Dobre, C. Doglioni, J. Dolejsi, Z. Dolezal, M. Donadelli, S. Donati, P. Dondero, J. Donini, J. Dopke, A. Doria, M. T. Dova, A. T. Doyle, E. Drechsler, M. Dris, Y. Du, J. Duarte-Campderros, E. Duchovni, G. Duckeck, O. A. Ducu, D. Duda, A. Dudarev, A. Chr. Dudder, E. M. Duffield, L. Duflot, M. Dührssen, M. Dumancic, M. Dunford, H. Duran Yildiz, M. Düren, A. Durglishvili, D. Duschinger, B. Dutta, M. Dyndal, C. Eckardt, K. M. Ecker, R. C. Edgar, N. C. Edwards, T. Eifert, G. Eigen, K. Einsweiler, T. Ekelof, M. El Kacimi, V. Ellajosyula, M. Ellert, S. Elles, F. Ellinghaus, A. A. Elliot, N. Ellis, J. Elmsheuser, M. Elsing, D. Emeliyanov, Y. Enari, O. C. Endner, J. S. Ennis, J. Erdmann, A. Ereditato, G. Ernis, J. Ernst, M. Ernst, S. Errede, E. Ertel, M. Escalier, H. Esch, C. Escobar, B. Esposito, A. I. Etienvre, E. Etzion, H. Evans, A. Ezhilov, M. Ezzi, F. Fabbri, L. Fabbri, G. Facini, R. M. Fakhrutdinov, S. Falciano, R. J. Falla, J. Faltova, Y. Fang, M. Fanti, A. Farbin, A. Farilla, C. Farina, E. M. Farina, T. Farooque, S. Farrell, S. M. Farrington, P. Farthouat, F. Fassi, P. Fassnacht, D. Fassouliotis, M. Faucci Giannelli, A. Favareto, W. J. Fawcett, L. Fayard, O. L. Fedin, W. Fedorko, S. Feigl, L. Feligioni, C. Feng, E. J. Feng, H. Feng, A. B. Fenyuk, L. Feremenga, P. Fernandez Martinez, S. Fernandez Perez, J. Ferrando, A. Ferrari, P. Ferrari, R. Ferrari, D. E. Ferreira de Lima, A. Ferrer, D. Ferrere, C. Ferretti, A. Ferretto Parodi, F. Fiedler, A. Filipčič, M. Filipuzzi, F. Filthaut, M. Fincke-Keeler, K. D. Finelli, M. C. N. Fiolhais, L. Fiorini, A. Firan, A. Fischer, C. Fischer, J. Fischer, W. C. Fisher, N. Flaschel, I. Fleck, P. Fleischmann, G. T. Fletcher, R. R. M. Fletcher, T. Flick, L. R. Flores Castillo, M. J. Flowerdew, G. T. Forcolin, A. Formica, A. Forti, A. G. Foster, D. Fournier, H. Fox, S. Fracchia, P. Francavilla, M. Franchini, D. Francis, L. Franconi, M. Franklin, M. Frate, M. Fraternali, D. Freeborn, S. M. Fressard-Batraneanu, F. Friedrich, D. Froidevaux, J. A. Frost, C. Fukunaga, E. Fullana Torregrosa, T. Fusayasu, J. Fuster, C. Gabaldon, O. Gabizon, A. Gabrielli, A. Gabrielli, G. P. Gach, S. Gadatsch, S. Gadomski, G. Gagliardi, L. G. Gagnon, P. Gagnon, C. Galea, B. Galhardo, E. J. Gallas, B. J. Gallop, P. Gallus, G. Galster, K. K. Gan, J. Gao, Y. Gao, Y. S. Gao, F. M. Garay Walls, C. García, J. E. García Navarro, M. Garcia-Sciveres, R. W. Gardner, N. Garelli, V. Garonne, A. Gascon Bravo, K. Gasnikova, C. Gatti, A. Gaudiello, G. Gaudio, L. Gauthier, I. L. Gavrilenko, C. Gay, G. Gaycken, E. N. Gazis, Z. Gecse, C. N. P. Gee, Ch. Geich-Gimbel, M. Geisen, M. P. Geisler, K. Gellerstedt, C. Gemme, M. H. Genest, C. Geng, S. Gentile, C. Gentsos, S. George, D. Gerbaudo, A. Gershon, S. Ghasemi, M. Ghneimat, B. Giacobbe, S. Giagu, P. Giannetti, B. Gibbard, S. M. Gibson, M. Gignac, M. Gilchriese, T. P. S. Gillam, D. Gillberg, G. Gilles, D. M. Gingrich, N. Giokaris, M. P. Giordani, F. M. Giorgi, F. M. Giorgi, P. F. Giraud, P. Giromini, D. Giugni, F. Giuli, C. Giuliani, M. Giulini, B. K. Gjelsten, S. Gkaitatzis, I. Gkialas, E. L. Gkougkousis, L. K. Gladilin, C. Glasman, J. Glatzer, P. C. F. Glaysher, A. Glazov, M. Goblirsch-Kolb, J. Godlewski, S. Goldfarb, T. Golling, D. Golubkov, A. Gomes, R. Gonçalo, J. Goncalves Pinto Firmino Da Costa, G. Gonella, L. Gonella, A. Gongadze, S. González de la Hoz, G. Gonzalez Parra, S. Gonzalez-Sevilla, L. Goossens, P. A. Gorbounov, H. A. Gordon, I. Gorelov, B. Gorini, E. Gorini, A. Gorišek, E. Gornicki, A. T. Goshaw, C. Gössling, M. I. Gostkin, C. R. Goudet, D. Goujdami, A. G. Goussiou, N. Govender, E. Gozani, L. Graber, I. Grabowska-Bold, P. O. J. Gradin, P. Grafström, J. Gramling, E. Gramstad, S. Grancagnolo, V. Gratchev, P. M. Gravila, H. M. Gray, E. Graziani, Z. D. Greenwood, C. Grefe, K. Gregersen, I. M. Gregor, P. Grenier, K. Grevtsov, J. Griffiths, A. A. Grillo, K. Grimm, S. Grinstein, Ph. Gris, J.-F. Grivaz, S. Groh, J. P. Grohs, E. Gross, J. Grosse-Knetter, G. C. Grossi, Z. J. Grout, L. Guan, W. Guan, J. Guenther, F. Guescini, D. Guest, O. Gueta, E. Guido, T. Guillemin, S. Guindon, U. Gul, C. Gumpert, J. Guo, Y. Guo, R. Gupta, S. Gupta, G. Gustavino, P. Gutierrez, N. G. Gutierrez Ortiz, C. Gutschow, C. Guyot, C. Gwenlan, C. B. Gwilliam, A. Haas, C. Haber, H. K. Hadavand, N. Haddad, A. Hadef, S. Hageböck, M. Hagihara, Z. Hajduk, H. Hakobyan, M. Haleem, J. Haley, G. Halladjian, G. D. Hallewell, K. Hamacher, P. Hamal, K. Hamano, A. Hamilton, G. N. Hamity, P. G. Hamnett, L. Han, K. Hanagaki, K. Hanawa, M. Hance, B. Haney, P. Hanke, R. Hanna, J. B. Hansen, J. D. Hansen, M. C. Hansen, P. H. Hansen, K. Hara, A. S. Hard, T. Harenberg, F. Hariri, S. Harkusha, R. D. Harrington, P. F. Harrison, F. Hartjes, N. M. Hartmann, M. Hasegawa, Y. Hasegawa, A. Hasib, S. Hassani, S. Haug, R. Hauser, L. Hauswald, M. Havranek, C. M. Hawkes, R. J. Hawkings, D. Hayakawa, D. Hayden, C. P. Hays, J. M. Hays, H. S. Hayward, S. J. Haywood, S. J. Head, T. Heck, V. Hedberg, L. Heelan, S. Heim, T. Heim, B. Heinemann, J. J. Heinrich, L. Heinrich, C. Heinz, J. Hejbal, L. Helary, S. Hellman, C. Helsens, J. Henderson, R. C. W. Henderson, Y. Heng, S. Henkelmann, A. M. Henriques Correia, S. Henrot-Versille, G. H. Herbert, H. Herde, V. Herget, Y. Hernández Jiménez, G. Herten, R. Hertenberger, L. Hervas, G. G. Hesketh, N. P. Hessey, J. W. Hetherly, R. Hickling, E. Higón-Rodriguez, E. Hill, J. C. Hill, K. H. Hiller, S. J. Hillier, I. Hinchliffe, E. Hines, R. R. Hinman, M. Hirose, D. Hirschbuehl, J. Hobbs, N. Hod, M. C. Hodgkinson, P. Hodgson, A. Hoecker, M. R. Hoeferkamp, F. Hoenig, D. Hohn, T. R. Holmes, M. Homann, T. Honda, T. M. Hong, B. H. Hooberman, W. H. Hopkins, Y. Horii, A. J. Horton, J-Y. Hostachy, S. Hou, A. Hoummada, J. Howarth, J. Hoya, M. Hrabovsky, I. Hristova, J. Hrivnac, T. Hryn’ova, A. Hrynevich, C. Hsu, P. J. Hsu, S.-C. Hsu, Q. Hu, S. Hu, Y. Huang, Z. Hubacek, F. Hubaut, F. Huegging, T. B. Huffman, E. W. Hughes, G. Hughes, M. Huhtinen, P. Huo, N. Huseynov, J. Huston, J. Huth, G. Iacobucci, G. Iakovidis, I. Ibragimov, L. Iconomidou-Fayard, E. Ideal, Z. Idrissi, P. Iengo, O. Igonkina, T. Iizawa, Y. Ikegami, M. Ikeno, Y. Ilchenko, D. Iliadis, N. Ilic, T. Ince, G. Introzzi, P. Ioannou, M. Iodice, K. Iordanidou, V. Ippolito, N. Ishijima, M. Ishino, M. Ishitsuka, R. Ishmukhametov, C. Issever, S. Istin, F. Ito, J. M. Iturbe Ponce, R. Iuppa, W. Iwanski, H. Iwasaki, J. M. Izen, V. Izzo, S. Jabbar, B. Jackson, P. Jackson, V. Jain, K. B. Jakobi, K. Jakobs, S. Jakobsen, T. Jakoubek, D. O. Jamin, D. K. Jana, R. Jansky, J. Janssen, M. Janus, G. Jarlskog, N. Javadov, T. Javůrek, F. Jeanneau, L. Jeanty, G.-Y. Jeng, D. Jennens, P. Jenni, C. Jeske, S. Jézéquel, H. Ji, J. Jia, H. Jiang, Y. Jiang, S. Jiggins, J. Jimenez Pena, S. Jin, A. Jinaru, O. Jinnouchi, H. Jivan, P. Johansson, K. A. Johns, W. J. Johnson, K. Jon-And, G. Jones, R. W. L. Jones, S. Jones, T. J. Jones, J. Jongmanns, P. M. Jorge, J. Jovicevic, X. Ju, A. Juste Rozas, M. K. Köhler, A. Kaczmarska, M. Kado, H. Kagan, M. Kagan, S. J. Kahn, T. Kaji, E. Kajomovitz, C. W. Kalderon, A. Kaluza, S. Kama, A. Kamenshchikov, N. Kanaya, S. Kaneti, L. Kanjir, V. A. Kantserov, J. Kanzaki, B. Kaplan, L. S. Kaplan, A. Kapliy, D. Kar, K. Karakostas, A. Karamaoun, N. Karastathis, M. J. Kareem, E. Karentzos, M. Karnevskiy, S. N. Karpov, Z. M. Karpova, K. Karthik, V. Kartvelishvili, A. N. Karyukhin, K. Kasahara, L. Kashif, R. D. Kass, A. Kastanas, Y. Kataoka, C. Kato, A. Katre, J. Katzy, K Kawade, K. Kawagoe, T. Kawamoto, G. Kawamura, V. F. Kazanin, R. Keeler, R. Kehoe, J. S. Keller, J. J. Kempster, H. Keoshkerian, O. Kepka, B. P. Kerševan, S. Kersten, R. A. Keyes, M. Khader, F. Khalil-zada, A. Khanov, A. G. Kharlamov, T. Kharlamova, T. J. Khoo, V. Khovanskiy, E. Khramov, J. Khubua, S. Kido, C. R. Kilby, H. Y. Kim, S. H. Kim, Y. K. Kim, N. Kimura, O. M. Kind, B. T. King, M. King, J. Kirk, A. E. Kiryunin, T. Kishimoto, D. Kisielewska, F. Kiss, K. Kiuchi, O. Kivernyk, E. Kladiva, M. H. Klein, M. Klein, U. Klein, K. Kleinknecht, P. Klimek, A. Klimentov, R. Klingenberg, J. A. Klinger, T. Klioutchnikova, E.-E. Kluge, P. Kluit, S. Kluth, J. Knapik, E. Kneringer, E. B. F. G. Knoops, A. Knue, A. Kobayashi, D. Kobayashi, T. Kobayashi, M. Kobel, M. Kocian, P. Kodys, N. M. Koehler, T. Koffas, E. Koffeman, T. Koi, H. Kolanoski, M. Kolb, I. Koletsou, A. A. Komar, Y. Komori, T. Kondo, N. Kondrashova, K. Köneke, A. C. König, T. Kono, R. Konoplich, N. Konstantinidis, R. Kopeliansky, S. Koperny, L. Köpke, A. K. Kopp, K. Korcyl, K. Kordas, A. Korn, A. A. Korol, I. Korolkov, E. V. Korolkova, O. Kortner, S. Kortner, T. Kosek, V. V. Kostyukhin, A. Kotwal, A. Kourkoumeli-Charalampidi, C. Kourkoumelis, V. Kouskoura, A. B. Kowalewska, R. Kowalewski, T. Z. Kowalski, C. Kozakai, W. Kozanecki, A. S. Kozhin, V. A. Kramarenko, G. Kramberger, D. Krasnopevtsev, M. W. Krasny, A. Krasznahorkay, A. Kravchenko, M. Kretz, J. Kretzschmar, K. Kreutzfeldt, P. Krieger, K. Krizka, K. Kroeninger, H. Kroha, J. Kroll, J. Kroseberg, J. Krstic, U. Kruchonak, H. Krüger, N. Krumnack, M. C. Kruse, M. Kruskal, T. Kubota, H. Kucuk, S. Kuday, J. T. Kuechler, S. Kuehn, A. Kugel, F. Kuger, A. Kuhl, T. Kuhl, V. Kukhtin, R. Kukla, Y. Kulchitsky, S. Kuleshov, M. Kuna, T. Kunigo, A. Kupco, H. Kurashige, Y. A. Kurochkin, V. Kus, E. S. Kuwertz, M. Kuze, J. Kvita, T. Kwan, D. Kyriazopoulos, A. La Rosa, J. L. La Rosa Navarro, L. La Rotonda, C. Lacasta, F. Lacava, J. Lacey, H. Lacker, D. Lacour, V. R. Lacuesta, E. Ladygin, R. Lafaye, B. Laforge, T. Lagouri, S. Lai, S. Lammers, W. Lampl, E. Lançon, U. Landgraf, M. P. J. Landon, M. C. Lanfermann, V. S. Lang, J. C. Lange, A. J. Lankford, F. Lanni, K. Lantzsch, A. Lanza, S. Laplace, C. Lapoire, J. F. Laporte, T. Lari, F. Lasagni Manghi, M. Lassnig, P. Laurelli, W. Lavrijsen, A. T. Law, P. Laycock, T. Lazovich, M. Lazzaroni, B. Le, O. Le Dortz, E. Le Guirriec, E. P. Le Quilleuc, M. LeBlanc, T. LeCompte, F. Ledroit-Guillon, C. A. Lee, S. C. Lee, L. Lee, B. Lefebvre, G. Lefebvre, M. Lefebvre, F. Legger, C. Leggett, A. Lehan, G. Lehmann Miotto, X. Lei, W. A. Leight, A. Leisos, A. G. Leister, M. A. L. Leite, R. Leitner, D. Lellouch, B. Lemmer, K. J. C. Leney, T. Lenz, B. Lenzi, R. Leone, S. Leone, C. Leonidopoulos, S. Leontsinis, G. Lerner, C. Leroy, A. A. J. Lesage, C. G. Lester, M. Levchenko, J. Levêque, D. Levin, L. J. Levinson, M. Levy, D. Lewis, A. M. Leyko, M. Leyton, B. Li, C. Li, H. Li, H. L. Li, L. Li, L. Li, Q. Li, S. Li, X. Li, Y. Li, Z. Liang, B. Liberti, A. Liblong, P. Lichard, K. Lie, J. Liebal, W. Liebig, A. Limosani, S. C. Lin, T. H. Lin, B. E. Lindquist, A. E. Lionti, E. Lipeles, A. Lipniacka, M. Lisovyi, T. M. Liss, A. Lister, A. M. Litke, B. Liu, D. Liu, H. Liu, H. Liu, J. Liu, J. B. Liu, K. Liu, L. Liu, M. Liu, M. Liu, Y. L. Liu, Y. Liu, M. Livan, A. Lleres, J. Llorente Merino, S. L. Lloyd, F. Lo Sterzo, E. M. Lobodzinska, P. Loch, W. S. Lockman, F. K. Loebinger, A. E. Loevschall-Jensen, K. M. Loew, A. Loginov, T. Lohse, K. Lohwasser, M. Lokajicek, B. A. Long, J. D. Long, R. E. Long, L. Longo, K. A. Looper, J. A. López, D. Lopez Mateos, B. Lopez Paredes, I. Lopez Paz, A. Lopez Solis, J. Lorenz, N. Lorenzo Martinez, M. Losada, P. J. Lösel, X. Lou, A. Lounis, J. Love, P. A. Love, H. Lu, N. Lu, H. J. Lubatti, C. Luci, A. Lucotte, C. Luedtke, F. Luehring, W. Lukas, L. Luminari, O. Lundberg, B. Lund-Jensen, P. M. Luzi, D. Lynn, R. Lysak, E. Lytken, V. Lyubushkin, H. Ma, L. L. Ma, Y. Ma, G. Maccarrone, A. Macchiolo, C. M. Macdonald, B. Maček, J. Machado Miguens, D. Madaffari, R. Madar, H. J. Maddocks, W. F. Mader, A. Madsen, J. Maeda, S. Maeland, T. Maeno, A. Maevskiy, E. Magradze, J. Mahlstedt, C. Maiani, C. Maidantchik, A. A. Maier, T. Maier, A. Maio, S. Majewski, Y. Makida, N. Makovec, B. Malaescu, Pa. Malecki, V. P. Maleev, F. Malek, U. Mallik, D. Malon, C. Malone, C. Malone, S. Maltezos, S. Malyukov, J. Mamuzic, G. Mancini, L. Mandelli, I. Mandić, J. Maneira, L. Manhaes de Andrade Filho, J. Manjarres Ramos, A. Mann, A. Manousos, B. Mansoulie, J. D. Mansour, R. Mantifel, M. Mantoani, S. Manzoni, L. Mapelli, G. Marceca, L. March, G. Marchiori, M. Marcisovsky, M. Marjanovic, D. E. Marley, F. Marroquim, S. P. Marsden, Z. Marshall, S. Marti-Garcia, B. Martin, T. A. Martin, V. J. Martin, B. Martin dit Latour, M. Martinez, V. I. Martinez Outschoorn, S. Martin-Haugh, V. S. Martoiu, A. C. Martyniuk, A. Marzin, L. Masetti, T. Mashimo, R. Mashinistov, J. Masik, A. L. Maslennikov, I. Massa, L. Massa, P. Mastrandrea, A. Mastroberardino, T. Masubuchi, P. Mättig, J. Mattmann, J. Maurer, S. J. Maxfield, D. A. Maximov, R. Mazini, S. M. Mazza, N. C. Mc Fadden, G. Mc Goldrick, S. P. Mc Kee, A. McCarn, R. L. McCarthy, T. G. McCarthy, L. I. McClymont, E. F. McDonald, J. A. Mcfayden, G. Mchedlidze, S. J. McMahon, R. A. McPherson, M. Medinnis, S. Meehan, S. Mehlhase, A. Mehta, K. Meier, C. Meineck, B. Meirose, D. Melini, B. R. Mellado Garcia, M. Melo, F. Meloni, A. Mengarelli, S. Menke, E. Meoni, S. Mergelmeyer, P. Mermod, L. Merola, C. Meroni, F. S. Merritt, A. Messina, J. Metcalfe, A. S. Mete, C. Meyer, C. Meyer, J.-P. Meyer, J. Meyer, H. Meyer Zu Theenhausen, F. Miano, R. P. Middleton, S. Miglioranzi, L. Mijović, G. Mikenberg, M. Mikestikova, M. Mikuž, M. Milesi, A. Milic, D. W. Miller, C. Mills, A. Milov, D. A. Milstead, A. A. Minaenko, Y. Minami, I. A. Minashvili, A. I. Mincer, B. Mindur, M. Mineev, Y. Minegishi, Y. Ming, L. M. Mir, K. P. Mistry, T. Mitani, J. Mitrevski, V. A. Mitsou, A. Miucci, P. S. Miyagawa, J. U. Mjörnmark, M. Mlynarikova, T. Moa, K. Mochizuki, S. Mohapatra, S. Molander, R. Moles-Valls, R. Monden, M. C. Mondragon, K. Mönig, J. Monk, E. Monnier, A. Montalbano, J. Montejo Berlingen, F. Monticelli, S. Monzani, R. W. Moore, N. Morange, D. Moreno, M. Moreno Llácer, P. Morettini, S. Morgenstern, D. Mori, T. Mori, M. Morii, M. Morinaga, V. Morisbak, S. Moritz, A. K. Morley, G. Mornacchi, J. D. Morris, S. S. Mortensen, L. Morvaj, M. Mosidze, J. Moss, K. Motohashi, R. Mount, E. Mountricha, E. J. W. Moyse, S. Muanza, R. D. Mudd, F. Mueller, J. Mueller, R. S. P. Mueller, T. Mueller, D. Muenstermann, P. Mullen, G. A. Mullier, F. J. Munoz Sanchez, J. A. Murillo Quijada, W. J. Murray, H. Musheghyan, M. Muškinja, A. G. Myagkov, M. Myska, B. P. Nachman, O. Nackenhorst, K. Nagai, R. Nagai, K. Nagano, Y. Nagasaka, K. Nagata, M. Nagel, E. Nagy, A. M. Nairz, Y. Nakahama, K. Nakamura, T. Nakamura, I. Nakano, H. Namasivayam, R. F. Naranjo Garcia, R. Narayan, D. I. Narrias Villar, I. Naryshkin, T. Naumann, G. Navarro, R. Nayyar, H. A. Neal, P. Yu. Nechaeva, T. J. Neep, A. Negri, M. Negrini, S. Nektarijevic, C. Nellist, A. Nelson, S. Nemecek, P. Nemethy, A. A. Nepomuceno, M. Nessi, M. S. Neubauer, M. Neumann, R. M. Neves, P. Nevski, P. R. Newman, D. H. Nguyen, T. Nguyen Manh, R. B. Nickerson, R. Nicolaidou, J. Nielsen, A. Nikiforov, V. Nikolaenko, I. Nikolic-Audit, K. Nikolopoulos, J. K. Nilsen, P. Nilsson, Y. Ninomiya, A. Nisati, R. Nisius, T. Nobe, M. Nomachi, I. Nomidis, T. Nooney, S. Norberg, M. Nordberg, N. Norjoharuddeen, O. Novgorodova, S. Nowak, M. Nozaki, L. Nozka, K. Ntekas, E. Nurse, F. Nuti, F. O’grady, D. C. O’Neil, A. A. O’Rourke, V. O’Shea, F. G. Oakham, H. Oberlack, T. Obermann, J. Ocariz, A. Ochi, I. Ochoa, J. P. Ochoa-Ricoux, S. Oda, S. Odaka, H. Ogren, A. Oh, S. H. Oh, C. C. Ohm, H. Ohman, H. Oide, H. Okawa, Y. Okumura, T. Okuyama, A. Olariu, L. F. Oleiro Seabra, S. A. Olivares Pino, D. Oliveira Damazio, A. Olszewski, J. Olszowska, A. Onofre, K. Onogi, P. U. E. Onyisi, M. J. Oreglia, Y. Oren, D. Orestano, N. Orlando, R. S. Orr, B. Osculati, R. Ospanov, G. Otero y Garzon, H. Otono, M. Ouchrif, F. Ould-Saada, A. Ouraou, K. P. Oussoren, Q. Ouyang, M. Owen, R. E. Owen, V. E. Ozcan, N. Ozturk, K. Pachal, A. Pacheco Pages, L. Pacheco Rodriguez, C. Padilla Aranda, M. Pagáčová, S. Pagan Griso, M. Paganini, F. Paige, P. Pais, K. Pajchel, G. Palacino, S. Palazzo, S. Palestini, M. Palka, D. Pallin, E. St. Panagiotopoulou, C. E. Pandini, J. G. Panduro Vazquez, P. Pani, S. Panitkin, D. Pantea, L. Paolozzi, Th. D. Papadopoulou, K. Papageorgiou, A. Paramonov, D. Paredes Hernandez, A. J. Parker, M. A. Parker, K. A. Parker, F. Parodi, J. A. Parsons, U. Parzefall, V. R. Pascuzzi, E. Pasqualucci, S. Passaggio, Fr. Pastore, G. Pásztor, S. Pataraia, J. R. Pater, T. Pauly, J. Pearce, B. Pearson, L. E. Pedersen, M. Pedersen, S. Pedraza Lopez, R. Pedro, S. V. Peleganchuk, O. Penc, C. Peng, H. Peng, J. Penwell, B. S. Peralva, M. M. Perego, D. V. Perepelitsa, E. Perez Codina, L. Perini, H. Pernegger, S. Perrella, R. Peschke, V. D. Peshekhonov, K. Peters, R. F. Y. Peters, B. A. Petersen, T. C. Petersen, E. Petit, A. Petridis, C. Petridou, P. Petroff, E. Petrolo, M. Petrov, F. Petrucci, N. E. Pettersson, A. Peyaud, R. Pezoa, P. W. Phillips, G. Piacquadio, E. Pianori, A. Picazio, E. Piccaro, M. Piccinini, M. A. Pickering, R. Piegaia, J. E. Pilcher, A. D. Pilkington, A. W. J. Pin, M. Pinamonti, J. L. Pinfold, A. Pingel, S. Pires, H. Pirumov, M. Pitt, L. Plazak, M. -A. Pleier, V. Pleskot, E. Plotnikova, P. Plucinski, D. Pluth, R. Poettgen, L. Poggioli, D. Pohl, G. Polesello, A. Poley, A. Policicchio, R. Polifka, A. Polini, C. S. Pollard, V. Polychronakos, K. Pommès, L. Pontecorvo, B. G. Pope, G. A. Popeneciu, A. Poppleton, S. Pospisil, K. Potamianos, I. N. Potrap, C. J. Potter, C. T. Potter, G. Poulard, J. Poveda, V. Pozdnyakov, M. E. Pozo Astigarraga, P. Pralavorio, A. Pranko, S. Prell, D. Price, L. E. Price, M. Primavera, S. Prince, K. Prokofiev, F. Prokoshin, S. Protopopescu, J. Proudfoot, M. Przybycien, D. Puddu, M. Purohit, P. Puzo, J. Qian, G. Qin, Y. Qin, A. Quadt, W. B. Quayle, M. Queitsch-Maitland, D. Quilty, S. Raddum, V. Radeka, V. Radescu, S. K. Radhakrishnan, P. Radloff, P. Rados, F. Ragusa, G. Rahal, J. A. Raine, S. Rajagopalan, M. Rammensee, C. Rangel-Smith, M. G. Ratti, F. Rauscher, S. Rave, T. Ravenscroft, I. Ravinovich, M. Raymond, A. L. Read, N. P. Readioff, M. Reale, D. M. Rebuzzi, A. Redelbach, G. Redlinger, R. Reece, R. G. Reed, K. Reeves, L. Rehnisch, J. Reichert, A. Reiss, C. Rembser, H. Ren, M. Rescigno, S. Resconi, O. L. Rezanova, P. Reznicek, R. Rezvani, R. Richter, S. Richter, E. Richter-Was, O. Ricken, M. Ridel, P. Rieck, C. J. Riegel, J. Rieger, O. Rifki, M. Rijssenbeek, A. Rimoldi, M. Rimoldi, L. Rinaldi, B. Ristić, E. Ritsch, I. Riu, F. Rizatdinova, E. Rizvi, C. Rizzi, S. H. Robertson, A. Robichaud-Veronneau, D. Robinson, J. E. M. Robinson, A. Robson, C. Roda, Y. Rodina, A. Rodriguez Perez, D. Rodriguez Rodriguez, S. Roe, C. S. Rogan, O. Røhne, A. Romaniouk, M. Romano, S. M. Romano Saez, E. Romero Adam, N. Rompotis, M. Ronzani, L. Roos, E. Ros, S. Rosati, K. Rosbach, P. Rose, N.-A. Rosien, V. Rossetti, E. Rossi, L. P. Rossi, J. H. N. Rosten, R. Rosten, M. Rotaru, I. Roth, J. Rothberg, D. Rousseau, A. Rozanov, Y. Rozen, X. Ruan, F. Rubbo, M. S. Rudolph, F. Rühr, A. Ruiz-Martinez, Z. Rurikova, N. A. Rusakovich, A. Ruschke, H. L. Russell, J. P. Rutherfoord, N. Ruthmann, Y. F. Ryabov, M. Rybar, G. Rybkin, S. Ryu, A. Ryzhov, G. F. Rzehorz, A. F. Saavedra, G. Sabato, S. Sacerdoti, H. F.-W. Sadrozinski, R. Sadykov, F. Safai Tehrani, P. Saha, M. Sahinsoy, M. Saimpert, T. Saito, H. Sakamoto, Y. Sakurai, G. Salamanna, A. Salamon, J. E. Salazar Loyola, D. Salek, P. H. Sales De Bruin, D. Salihagic, A. Salnikov, J. Salt, D. Salvatore, F. Salvatore, A. Salvucci, A. Salzburger, D. Sammel, D. Sampsonidis, A. Sanchez, J. Sánchez, V. Sanchez Martinez, H. Sandaker, R. L. Sandbach, H. G. Sander, M. Sandhoff, C. Sandoval, D. P. C. Sankey, M. Sannino, A. Sansoni, C. Santoni, R. Santonico, H. Santos, I. Santoyo Castillo, K. Sapp, A. Sapronov, J. G. Saraiva, B. Sarrazin, O. Sasaki, K. Sato, E. Sauvan, G. Savage, P. Savard, N. Savic, C. Sawyer, L. Sawyer, J. Saxon, C. Sbarra, A. Sbrizzi, T. Scanlon, D. A. Scannicchio, M. Scarcella, V. Scarfone, J. Schaarschmidt, P. Schacht, B. M. Schachtner, D. Schaefer, L. Schaefer, R. Schaefer, J. Schaeffer, S. Schaepe, S. Schaetzel, U. Schäfer, A. C. Schaffer, D. Schaile, R. D. Schamberger, V. Scharf, V. A. Schegelsky, D. Scheirich, M. Schernau, C. Schiavi, S. Schier, C. Schillo, M. Schioppa, S. Schlenker, K. R. Schmidt-Sommerfeld, K. Schmieden, C. Schmitt, S. Schmitt, S. Schmitz, B. Schneider, U. Schnoor, L. Schoeffel, A. Schoening, B. D. Schoenrock, E. Schopf, M. Schott, J. F. P. Schouwenberg, J. Schovancova, S. Schramm, M. Schreyer, N. Schuh, A. Schulte, M. J. Schultens, H.-C. Schultz-Coulon, H. Schulz, M. Schumacher, B. A. Schumm, Ph. Schune, A. Schwartzman, T. A. Schwarz, H. Schweiger, Ph. Schwemling, R. Schwienhorst, J. Schwindling, T. Schwindt, G. Sciolla, F. Scuri, F. Scutti, J. Searcy, P. Seema, S. C. Seidel, A. Seiden, F. Seifert, J. M. Seixas, G. Sekhniaidze, K. Sekhon, S. J. Sekula, D. M. Seliverstov, N. Semprini-Cesari, C. Serfon, L. Serin, L. Serkin, M. Sessa, R. Seuster, H. Severini, T. Sfiligoj, F. Sforza, A. Sfyrla, E. Shabalina, N. W. Shaikh, L. Y. Shan, R. Shang, J. T. Shank, M. Shapiro, P. B. Shatalov, K. Shaw, S. M. Shaw, A. Shcherbakova, C. Y. Shehu, P. Sherwood, L. Shi, S. Shimizu, C. O. Shimmin, M. Shimojima, S. Shirabe, M. Shiyakova, A. Shmeleva, D. Shoaleh Saadi, M. J. Shochet, S. Shojaii, D. R. Shope, S. Shrestha, E. Shulga, M. A. Shupe, P. Sicho, A. M. Sickles, P. E. Sidebo, O. Sidiropoulou, D. Sidorov, A. Sidoti, F. Siegert, Dj. Sijacki, J. Silva, S. B. Silverstein, V. Simak, Lj. Simic, S. Simion, E. Simioni, B. Simmons, D. Simon, M. Simon, P. Sinervo, N. B. Sinev, M. Sioli, G. Siragusa, I. Siral, S. Yu. Sivoklokov, J. Sjölin, M. B. Skinner, H. P. Skottowe, P. Skubic, M. Slater, T. Slavicek, M. Slawinska, K. Sliwa, R. Slovak, V. Smakhtin, B. H. Smart, L. Smestad, J. Smiesko, S. Yu. Smirnov, Y. Smirnov, L. N. Smirnova, O. Smirnova, M. N. K. Smith, R. W. Smith, M. Smizanska, K. Smolek, A. A. Snesarev, I. M. Snyder, S. Snyder, R. Sobie, F. Socher, A. Soffer, D. A. Soh, G. Sokhrannyi, C. A. Solans Sanchez, M. Solar, E. Yu. Soldatov, U. Soldevila, A. A. Solodkov, A. Soloshenko, O. V. Solovyanov, V. Solovyev, P. Sommer, H. Son, H. Y. Song, A. Sood, A. Sopczak, V. Sopko, V. Sorin, D. Sosa, C. L. Sotiropoulou, R. Soualah, A. M. Soukharev, D. South, B. C. Sowden, S. Spagnolo, M. Spalla, M. Spangenberg, F. Spanò, D. Sperlich, F. Spettel, R. Spighi, G. Spigo, L. A. Spiller, M. Spousta, R. D. St. Denis, A. Stabile, R. Stamen, S. Stamm, E. Stanecka, R. W. Stanek, C. Stanescu, M. Stanescu-Bellu, M. M. Stanitzki, S. Stapnes, E. A. Starchenko, G. H. Stark, J. Stark, P. Staroba, P. Starovoitov, S. Stärz, R. Staszewski, P. Steinberg, B. Stelzer, H. J. Stelzer, O. Stelzer-Chilton, H. Stenzel, G. A. Stewart, J. A. Stillings, M. C. Stockton, M. Stoebe, G. Stoicea, P. Stolte, S. Stonjek, A. R. Stradling, A. Straessner, M. E. Stramaglia, J. Strandberg, S. Strandberg, A. Strandlie, M. Strauss, P. Strizenec, R. Ströhmer, D. M. Strom, R. Stroynowski, A. Strubig, S. A. Stucci, B. Stugu, N. A. Styles, D. Su, J. Su, S. Suchek, Y. Sugaya, M. Suk, V. V. Sulin, S. Sultansoy, T. Sumida, S. Sun, X. Sun, J. E. Sundermann, K. Suruliz, G. Susinno, M. R. Sutton, S. Suzuki, M. Svatos, M. Swiatlowski, I. Sykora, T. Sykora, D. Ta, C. Taccini, K. Tackmann, J. Taenzer, A. Taffard, R. Tafirout, N. Taiblum, H. Takai, R. Takashima, T. Takeshita, Y. Takubo, M. Talby, A. A. Talyshev, K. G. Tan, J. Tanaka, M. Tanaka, R. Tanaka, S. Tanaka, R. Tanioka, B. B. Tannenwald, S. Tapia Araya, S. Tapprogge, S. Tarem, G. F. Tartarelli, P. Tas, M. Tasevsky, T. Tashiro, E. Tassi, A. Tavares Delgado, Y. Tayalati, A. C. Taylor, G. N. Taylor, P. T. E. Taylor, W. Taylor, F. A. Teischinger, P. Teixeira-Dias, K. K. Temming, D. Temple, H. Ten Kate, P. K. Teng, J. J. Teoh, F. Tepel, S. Terada, K. Terashi, J. Terron, S. Terzo, M. Testa, R. J. Teuscher, T. Theveneaux-Pelzer, J. P. Thomas, J. Thomas-Wilsker, E. N. Thompson, P. D. Thompson, A. S. Thompson, L. A. Thomsen, E. Thomson, M. Thomson, M. J. Tibbetts, R. E. Ticse Torres, V. O. Tikhomirov, Yu. A. Tikhonov, S. Timoshenko, P. Tipton, S. Tisserant, K. Todome, T. Todorov, S. Todorova-Nova, J. Tojo, S. Tokár, K. Tokushuku, E. Tolley, L. Tomlinson, M. Tomoto, L. Tompkins, K. Toms, B. Tong, P. Tornambe, E. Torrence, H. Torres, E. Torró Pastor, J. Toth, F. Touchard, D. R. Tovey, T. Trefzger, A. Tricoli, I. M. Trigger, S. Trincaz-Duvoid, M. F. Tripiana, W. Trischuk, B. Trocmé, A. Trofymov, C. Troncon, M. Trottier-McDonald, M. Trovatelli, L. Truong, M. Trzebinski, A. Trzupek, J. C.-L. Tseng, P. V. Tsiareshka, G. Tsipolitis, N. Tsirintanis, S. Tsiskaridze, V. Tsiskaridze, E. G. Tskhadadze, K. M. Tsui, I. I. Tsukerman, V. Tsulaia, S. Tsuno, D. Tsybychev, Y. Tu, A. Tudorache, V. Tudorache, A. N. Tuna, S. A. Tupputi, S. Turchikhin, D. Turecek, D. Turgeman, R. Turra, P. M. Tuts, M. Tyndel, G. Ucchielli, I. Ueda, M. Ughetto, F. Ukegawa, G. Unal, A. Undrus, G. Unel, F. C. Ungaro, Y. Unno, C. Unverdorben, J. Urban, P. Urquijo, P. Urrejola, G. Usai, L. Vacavant, V. Vacek, B. Vachon, C. Valderanis, E. Valdes Santurio, N. Valencic, S. Valentinetti, A. Valero, L. Valery, S. Valkar, J. A. Valls Ferrer, W. Van Den Wollenberg, P. C. Van Der Deijl, H. van der Graaf, N. van Eldik, P. van Gemmeren, J. Van Nieuwkoop, I. van Vulpen, M. C. van Woerden, M. Vanadia, W. Vandelli, R. Vanguri, A. Vaniachine, P. Vankov, G. Vardanyan, R. Vari, E. W. Varnes, T. Varol, D. Varouchas, A. Vartapetian, K. E. Varvell, J. G. Vasquez, G. A. Vasquez, F. Vazeille, T. Vazquez Schroeder, J. Veatch, V. Veeraraghavan, L. M. Veloce, F. Veloso, S. Veneziano, A. Ventura, M. Venturi, N. Venturi, A. Venturini, V. Vercesi, M. Verducci, W. Verkerke, J. C. Vermeulen, A. Vest, M. C. Vetterli, O. Viazlo, I. Vichou, T. Vickey, O. E. Vickey Boeriu, G. H. A. Viehhauser, S. Viel, L. Vigani, M. Villa, M. Villaplana Perez, E. Vilucchi, M. G. Vincter, V. B. Vinogradov, C. Vittori, I. Vivarelli, S. Vlachos, M. Vlasak, M. Vogel, P. Vokac, G. Volpi, M. Volpi, H. von der Schmitt, E. von Toerne, V. Vorobel, K. Vorobev, M. Vos, R. Voss, J. H. Vossebeld, N. Vranjes, M. Vranjes Milosavljevic, V. Vrba, M. Vreeswijk, R. Vuillermet, I. Vukotic, Z. Vykydal, P. Wagner, W. Wagner, H. Wahlberg, S. Wahrmund, J. Wakabayashi, J. Walder, R. Walker, W. Walkowiak, V. Wallangen, C. Wang, C. Wang, F. Wang, H. Wang, H. Wang, J. Wang, J. Wang, K. Wang, R. Wang, S. M. Wang, T. Wang, T. Wang, W. Wang, X. Wang, C. Wanotayaroj, A. Warburton, C. P. Ward, D. R. Wardrope, A. Washbrook, P. M. Watkins, A. T. Watson, M. F. Watson, G. Watts, S. Watts, B. M. Waugh, S. Webb, M. S. Weber, S. W. Weber, S. A. Weber, J. S. Webster, A. R. Weidberg, B. Weinert, J. Weingarten, C. Weiser, H. Weits, P. S. Wells, T. Wenaus, T. Wengler, S. Wenig, N. Wermes, M. Werner, M. D. Werner, P. Werner, M. Wessels, J. Wetter, K. Whalen, N. L. Whallon, A. M. Wharton, A. White, M. J. White, R. White, D. Whiteson, F. J. Wickens, W. Wiedenmann, M. Wielers, C. Wiglesworth, L. A. M. Wiik-Fuchs, A. Wildauer, F. Wilk, H. G. Wilkens, H. H. Williams, S. Williams, C. Willis, S. Willocq, J. A. Wilson, I. Wingerter-Seez, F. Winklmeier, O. J. Winston, B. T. Winter, M. Wittgen, J. Wittkowski, T. M. H. Wolf, M. W. Wolter, H. Wolters, S. D. Worm, B. K. Wosiek, J. Wotschack, M. J. Woudstra, K. W. Wozniak, M. Wu, M. Wu, S. L. Wu, X. Wu, Y. Wu, T. R. Wyatt, B. M. Wynne, S. Xella, D. Xu, L. Xu, T. Xu, B. Yabsley, S. Yacoob, D. Yamaguchi, Y. Yamaguchi, A. Yamamoto, S. Yamamoto, T. Yamanaka, K. Yamauchi, Y. Yamazaki, Z. Yan, H. Yang, H. Yang, Y. Yang, Z. Yang, W.-M. Yao, Y. C. Yap, Y. Yasu, E. Yatsenko, K. H. Yau Wong, J. Ye, S. Ye, I. Yeletskikh, A. L. Yen, E. Yildirim, K. Yorita, R. Yoshida, K. Yoshihara, C. Young, C. J. S. Young, S. Youssef, D. R. Yu, J. Yu, J. M. Yu, J. Yu, L. Yuan, S. P. Y. Yuen, I. Yusuff, B. Zabinski, R. Zaidan, A. M. Zaitsev, N. Zakharchuk, J. Zalieckas, A. Zaman, S. Zambito, L. Zanello, D. Zanzi, C. Zeitnitz, M. Zeman, A. Zemla, J. C. Zeng, Q. Zeng, K. Zengel, O. Zenin, T. Ženiš, D. Zerwas, D. Zhang, F. Zhang, G. Zhang, H. Zhang, J. Zhang, L. Zhang, R. Zhang, R. Zhang, X. Zhang, Z. Zhang, X. Zhao, Y. Zhao, Z. Zhao, A. Zhemchugov, J. Zhong, B. Zhou, C. Zhou, L. Zhou, L. Zhou, M. Zhou, N. Zhou, C. G. Zhu, H. Zhu, J. Zhu, Y. Zhu, X. Zhuang, K. Zhukov, A. Zibell, D. Zieminska, N. I. Zimine, C. Zimmermann, S. Zimmermann, Z. Zinonos, M. Zinser, M. Ziolkowski, L. Živković, G. Zobernig, A. Zoccoli, M. zur Nedden, L. Zwalinski, ATLAS Collaboration

**Affiliations:** 1 Department of Physics, University of Adelaide, Adelaide, Australia; 2 Physics Department, SUNY Albany, Albany, NY USA; 3 Department of Physics, University of Alberta, Edmonton, AB Canada; 4 Department of Physics, Ankara University, Ankara, Turkey; 5 Istanbul Aydin University, Istanbul, Turkey; 6 Division of Physics, TOBB University of Economics and Technology, Ankara, Turkey; 7 LAPP, CNRS/IN2P3 and Université Savoie Mont Blanc, Annecy-le-Vieux, France; 8 High Energy Physics Division, Argonne National Laboratory, Argonne, IL USA; 9 Department of Physics, University of Arizona, Tucson, AZ USA; 10 Department of Physics, The University of Texas at Arlington, Arlington, TX USA; 11 Physics Department, University of Athens, Athens, Greece; 12 Physics Department, National Technical University of Athens, Zografou, Greece; 13 Department of Physics, The University of Texas at Austin, Austin, TX USA; 14 Institute of Physics, Azerbaijan Academy of Sciences, Baku, Azerbaijan; 15 Institut de Física d’Altes Energies (IFAE), The Barcelona Institute of Science and Technology, Barcelona, Spain; 16 Institute of Physics, University of Belgrade, Belgrade, Serbia; 17 Department for Physics and Technology, University of Bergen, Bergen, Norway; 18 Physics Division, Lawrence Berkeley National Laboratory and University of California, Berkeley, CA USA; 19 Department of Physics, Humboldt University, Berlin, Germany; 20 Albert Einstein Center for Fundamental Physics and Laboratory for High Energy Physics, University of Bern, Bern, Switzerland; 21 School of Physics and Astronomy, University of Birmingham, Birmingham, UK; 22 Department of Physics, Bogazici University, Istanbul, Turkey; 23 Department of Physics Engineering, Gaziantep University, Gaziantep, Turkey; 24 Istanbul Bilgi University, Faculty of Engineering and Natural Sciences, Istanbul, Turkey; 25 Bahcesehir University, Faculty of Engineering and Natural Sciences, Istanbul, Turkey; 26 Centro de Investigaciones, Universidad Antonio Narino, Bogota, Colombia; 27 INFN Sezione di Bologna, Bologna, Italy; 28 Dipartimento di Fisica e Astronomia, Università di Bologna, Bologna, Italy; 29 Physikalisches Institut, University of Bonn, Bonn, Germany; 30 Department of Physics, Boston University, Boston, MA USA; 31 Department of Physics, Brandeis University, Waltham, MA USA; 32 Universidade Federal do Rio De Janeiro COPPE/EE/IF, Rio de Janeiro, Brazil; 33 Electrical Circuits Department, Federal University of Juiz de Fora (UFJF), Juiz de Fora, Brazil; 34 Federal University of Sao Joao del Rei (UFSJ), Sao Joao del Rei, Brazil; 35 Instituto de Fisica, Universidade de Sao Paulo, São Paulo, Brazil; 36 Physics Department, Brookhaven National Laboratory, Upton, NY USA; 37 Transilvania University of Brasov, Brasov, Romania; 38 National Institute of Physics and Nuclear Engineering, Bucharest, Romania; 39 Physics Department, National Institute for Research and Development of Isotopic and Molecular Technologies, Cluj Napoca, Romania; 40 University Politehnica Bucharest, Bucharest, Romania; 41 West University in Timisoara, Timisoara, Romania; 42 Departamento de Física, Universidad de Buenos Aires, Buenos Aires, Argentina; 43 Cavendish Laboratory, University of Cambridge, Cambridge, UK; 44 Department of Physics, Carleton University, Ottawa, ON Canada; 45 CERN, Geneva, Switzerland; 46 Enrico Fermi Institute, University of Chicago, Chicago, IL USA; 47 Departamento de Física, Pontificia Universidad Católica de Chile, Santiago, Chile; 48 Departamento de Física, Universidad Técnica Federico Santa María, Valparaisío, Chile; 49 Institute of High Energy Physics, Chinese Academy of Sciences, Beijing, China; 50 Department of Physics, Nanjing University, Jiangsu, China; 51 Physics Department, Tsinghua University, Beijing, 100084 China; 52 Laboratoire de Physique Corpusculaire, Clermont Université ,and Université Blaise Pascal and CNRS/IN2P3, Clermont-Ferrand, France; 53 Nevis Laboratory, Columbia University, Irvington, NY USA; 54 Niels Bohr Institute, University of Copenhagen, Copenhagen, Denmark; 55 INFN Gruppo Collegato di Cosenza, Laboratori Nazionali di Frascati, Frascati, Italy; 56 Dipartimento di Fisica, Università della Calabria, Rende, Italy; 57 Faculty of Physics and Applied Computer Science, AGH University of Science and Technology, Kraków, Poland; 58 Marian Smoluchowski Institute of Physics, Jagiellonian University, Kraków, Poland; 59 Institute of Nuclear Physics, Polish Academy of Sciences, Kraków, Poland; 60 Physics Department, Southern Methodist University, Dallas, TX USA; 61 Physics Department, University of Texas at Dallas, Richardson, TX USA; 62 DESY, Hamburg and Zeuthen, Germany; 63 Lehrstuhl für Experimentelle Physik IV, Technische Universität Dortmund, Dortmund, Germany; 64 Institut für Kern- und Teilchenphysik, Technische Universität Dresden, Dresden, Germany; 65 Department of Physics, Duke University, Durham, NC USA; 66 SUPA - School of Physics and Astronomy, University of Edinburgh, Edinburgh, UK; 67 INFN Laboratori Nazionali di Frascati, Frascati, Italy; 68 Fakultät für Mathematik und Physik, Albert-Ludwigs-Universität, Freiburg, Germany; 69 Section de Physique, Université de Genève, Geneva, Switzerland; 70 INFN Sezione di Genova, Genoa, Italy; 71 Dipartimento di Fisica, Università di Genova, Genoa, Italy; 72 E. Andronikashvili Institute of Physics, Iv. Javakhishvili Tbilisi State University, Tbilisi, Georgia; 73 High Energy Physics Institute, Tbilisi State University, Tbilisi, Georgia; 74 II Physikalisches Institut, Justus-Liebig-Universität Giessen, Giessen, Germany; 75 SUPA - School of Physics and Astronomy, University of Glasgow, Glasgow, UK; 76 II Physikalisches Institut, Georg-August-Universität, Göttingen, Germany; 77 Laboratoire de Physique Subatomique et de Cosmologie, Université Grenoble-Alpes, CNRS/IN2P3, Grenoble, France; 78 Laboratory for Particle Physics and Cosmology, Harvard University, Cambridge, MA USA; 79 Department of Modern Physics, University of Science and Technology of China, Anhui, China; 80 Kirchhoff-Institut für Physik, Ruprecht-Karls-Universität Heidelberg, Heidelberg, Germany; 81 Physikalisches Institut, Ruprecht-Karls-Universität Heidelberg, Heidelberg, Germany; 82 ZITI Institut für technische Informatik, Ruprecht-Karls-Universität Heidelberg, Mannheim, Germany; 83 Faculty of Applied Information Science, Hiroshima Institute of Technology, Hiroshima, Japan; 84 Department of Physics, The Chinese University of Hong Kong, Shatin, N.T. Hong Kong; 85 Department of Physics, The University of Hong Kong, Hong Kong, China; 86 Department of Physics, The Hong Kong University of Science and Technology, Clear Water Bay, Kowloon, Hong Kong, China; 87 Department of Physics, Indiana University, Bloomington, IN United States of America; 88 Institut für Astro- und Teilchenphysik, Leopold-Franzens-Universität, Innsbruck, Austria; 89 University of Iowa, Iowa City, IA USA; 90 Department of Physics and Astronomy, Iowa State University, Ames, IA USA; 91 Joint Institute for Nuclear Research, JINR Dubna, Dubna, Russia; 92 KEK, High Energy Accelerator Research Organization, Tsukuba, Japan; 93 Graduate School of Science, Kobe University, Kobe, Japan; 94 Faculty of Science, Kyoto University, Kyoto, Japan; 95 Kyoto University of Education, Kyoto, Japan; 96 Department of Physics, Kyushu University, Fukuoka, Japan; 97 Instituto de Física La Plata, Universidad Nacional de La Plata and CONICET, La Plata, Argentina; 98 Physics Department, Lancaster University, Lancaster, UK; 99 INFN Sezione di Lecce, Lecce, Italy; 100 Dipartimento di Matematica e Fisica, Università del Salento, Lecce, Italy; 101 Oliver Lodge Laboratory, University of Liverpool, Liverpool, UK; 102 Department of Physics, Jožef Stefan Institute, University of Ljubljana, Ljubljana, Slovenia; 103 School of Physics and Astronomy, Queen Mary University of London, London, UK; 104 Department of Physics, Royal Holloway University of London, Surrey, UK; 105 Department of Physics and Astronomy, University College London, London, UK; 106 Louisiana Tech University, Ruston, LA USA; 107 Laboratoire de Physique Nucléaire et de Hautes Energies, UPMC and Université Paris-Diderot and CNRS/IN2P3, Paris, France; 108 Fysiska institutionen, Lunds universitet, Lund, Sweden; 109 Departamento de Fisica Teorica C-15, Universidad Autonoma de Madrid, Madrid, Spain; 110 Institut für Physik, Universität Mainz, Mainz, Germany; 111 School of Physics and Astronomy, University of Manchester, Manchester, UK; 112 CPPM, Aix-Marseille Université and CNRS/IN2P3, Marseille, France; 113 Department of Physics, University of Massachusetts, Amherst, MA USA; 114 Department of Physics, McGill University, Montreal, QC Canada; 115 School of Physics, University of Melbourne, Victoria, Australia; 116 Department of Physics, The University of Michigan, Ann Arbor, MI USA; 117 Department of Physics and Astronomy, Michigan State University, East Lansing, MI USA; 118 INFN Sezione di Milano, Milan, Italy; 119 Dipartimento di Fisica, Università di Milano, Milan, Italy; 120 B.I. Stepanov Institute of Physics, National Academy of Sciences of Belarus, Minsk, Republic of Belarus; 121 National Scientific and Educational Centre for Particle and High Energy Physics, Minsk, Republic of Belarus; 122 Group of Particle Physics, University of Montreal, Montreal, QC Canada; 123 P.N. Lebedev Physical Institute of the Russian Academy of Sciences, Moscow, Russia; 124 Institute for Theoretical and Experimental Physics (ITEP), Moscow, Russia; 125 National Research Nuclear University MEPhI, Moscow, Russia; 126 D.V. Skobeltsyn Institute of Nuclear Physics, M.V. Lomonosov Moscow State University, Moscow, Russia; 127 Fakultät für Physik, Ludwig-Maximilians-Universität München, Munich, Germany; 128 Max-Planck-Institut für Physik (Werner-Heisenberg-Institut), Munich, Germany; 129 Nagasaki Institute of Applied Science, Nagasaki, Japan; 130 Graduate School of Science and Kobayashi-Maskawa Institute, Nagoya University, Nagoya, Japan; 131 INFN Sezione di Napoli, Naples, Italy; 132 Dipartimento di Fisica, Università di Napoli, Naples, Italy; 133 Department of Physics and Astronomy, University of New Mexico, Albuquerque, NM USA; 134 Institute for Mathematics, Astrophysics and Particle Physics, Radboud University Nijmegen/Nikhef, Nijmegen, The Netherlands; 135 Nikhef National Institute for Subatomic Physics and University of Amsterdam, Amsterdam, The Netherlands; 136 Department of Physics, Northern Illinois University, DeKalb, IL USA; 137 Budker Institute of Nuclear Physics, SB RAS, Novosibirsk, Russia; 138 Department of Physics, New York University, New York, NY USA; 139 Ohio State University, Columbus, OH USA; 140 Faculty of Science, Okayama University, Okayama, Japan; 141 Homer L. Dodge Department of Physics and Astronomy, University of Oklahoma, Norman, OK USA; 142 Department of Physics, Oklahoma State University, Stillwater, OK USA; 143 Palacký University, RCPTM, Olomouc, Czech Republic; 144 Center for High Energy Physics, University of Oregon, Eugene, OR USA; 145 LAL, Univ. Paris-Sud, CNRS/IN2P3, Université Paris-Saclay, Orsay, France; 146 Graduate School of Science, Osaka University, Osaka, Japan; 147 Department of Physics, University of Oslo, Oslo, Norway; 148 Department of Physics, Oxford University, Oxford, UK; 149 INFN Sezione di Pavia, Pavia, Italy; 150 Dipartimento di Fisica, Università di Pavia, Pavia, Italy; 151 Department of Physics, University of Pennsylvania, Philadelphia, PA USA; 152 National Research Centre “Kurchatov Institute” B.P.Konstantinov Petersburg Nuclear Physics Institute, St. Petersburg, Russia; 153 INFN Sezione di Pisa, Pisa, Italy; 154 Dipartimento di Fisica E. Fermi, Università di Pisa, Pisa, Italy; 155 Department of Physics and Astronomy, University of Pittsburgh, Pittsburgh, PA USA; 156 Laboratório de Instrumentação e Física Experimental de Partículas-LIP, Lisbon, Portugal; 157 Faculdade de Ciências, Universidade de Lisboa, Lisbon, Portugal; 158 Department of Physics, University of Coimbra, Coimbra, Portugal; 159 Centro de Física Nuclear da Universidade de Lisboa, Lisbon, Portugal; 160 Departamento de Fisica, Universidade do Minho, Braga, Portugal; 161 Departamento de Fisica Teorica y del Cosmos and CAFPE, Universidad de Granada, Granada, Spain; 162 Dep Fisica and CEFITEC of Faculdade de Ciencias e Tecnologia, Universidade Nova de Lisboa, Caparica, Portugal; 163 Institute of Physics, Academy of Sciences of the Czech Republic, Prague, Czech Republic; 164 Czech Technical University in Prague, Prague, Czech Republic; 165 Faculty of Mathematics and Physics, Charles University in Prague, Prague, Czech Republic; 166 State Research Center Institute for High Energy Physics (Protvino), NRC KI, Protvino, Russia; 167 Particle Physics Department, Rutherford Appleton Laboratory, Didcot, UK; 168 INFN Sezione di Roma, Rome, Italy; 169 Dipartimento di Fisica, Sapienza Università di Roma, Rome, Italy; 170 INFN Sezione di Roma Tor Vergata, Rome, Italy; 171 Dipartimento di Fisica, Università di Roma Tor Vergata, Rome, Italy; 172 INFN Sezione di Roma Tre, Rome, Italy; 173 Dipartimento di Matematica e Fisica, Università Roma Tre, Rome, Italy; 174 Faculté des Sciences Ain Chock, Réseau Universitaire de Physique des Hautes Energies-Université Hassan II, Casablanca, Morocco; 175 Centre National de l’Energie des Sciences Techniques Nucleaires, Rabat, Morocco; 176 Faculté des Sciences Semlalia, Université Cadi Ayyad, LPHEA-Marrakech, Marrakech, Morocco; 177 Faculté des Sciences, Université Mohamed Premier and LPTPM, Oujda, Morocco; 178 Faculté des Sciences, Université Mohammed V, Rabat, Morocco; 179 DSM/IRFU (Institut de Recherches sur les Lois Fondamentales de l’Univers), CEA Saclay (Commissariat à l’Energie Atomique et aux Energies Alternatives), Gif-sur-Yvette, France; 180 Santa Cruz Institute for Particle Physics, University of California Santa Cruz, Santa Cruz, CA USA; 181 Department of Physics, University of Washington, Seattle, WA USA; 182 School of Physics, Shandong University, Shandong, China; 183 Department of Physics and Astronomy,Shanghai Key Laboratory for Particle Physics and Cosmology, Shanghai Jiao Tong University (also affiliated with PKU-CHEP), Shanghai, China; 184 Department of Physics and Astronomy, University of Sheffield, Sheffield, UK; 185 Department of Physics, Shinshu University, Nagano, Japan; 186 Fachbereich Physik, Universität Siegen, Siegen, Germany; 187 Department of Physics, Simon Fraser University, Burnaby, BC Canada; 188 SLAC National Accelerator Laboratory, Stanford, CA USA; 189 Faculty of Mathematics, Physics and Informatics, Comenius University, Bratislava, Slovak Republic; 190 Department of Subnuclear Physics, Institute of Experimental Physics of the Slovak Academy of Sciences, Kosice, Slovak Republic; 191 Department of Physics, University of Cape Town, Cape Town, South Africa; 192 Department of Physics, University of Johannesburg, Johannesburg, South Africa; 193 School of Physics, University of the Witwatersrand, Johannesburg, South Africa; 194 Department of Physics, Stockholm University, Stockholm, Sweden; 195 The Oskar Klein Centre, Stockholm, Sweden; 196 Physics Department, Royal Institute of Technology, Stockholm, Sweden; 197 Departments of Physics and Astronomy and Chemistry, Stony Brook University, Stony Brook, NY USA; 198 Department of Physics and Astronomy, University of Sussex, Brighton, UK; 199 School of Physics, University of Sydney, Sydney, Australia; 200 Institute of Physics, Academia Sinica, Taipei, Taiwan; 201 Department of Physics, Technion: Israel Institute of Technology, Haifa, Israel; 202 Raymond and Beverly Sackler School of Physics and Astronomy, Tel Aviv University, Tel Aviv, Israel; 203 Department of Physics, Aristotle University of Thessaloniki, Thessaloníki, Greece; 204 International Center for Elementary Particle Physics and Department of Physics, The University of Tokyo, Tokyo, Japan; 205 Graduate School of Science and Technology, Tokyo Metropolitan University, Tokyo, Japan; 206 Department of Physics, Tokyo Institute of Technology, Tokyo, Japan; 207 Tomsk State University, Tomsk, Russia Russia; 208 Department of Physics, University of Toronto, Toronto, ON Canada; 209 INFN-TIFPA, Trento, Italy; 210 University of Trento, Trento, Italy; 211 TRIUMF, Vancouver, BC Canada; 212 Department of Physics and Astronomy, York University, Toronto, ON Canada; 213 Faculty of Pure and Applied Sciences, and Center for Integrated Research in Fundamental Science and Engineering, University of Tsukuba, Tsukuba, Japan; 214 Department of Physics and Astronomy, Tufts University, Medford, MA USA; 215 Department of Physics and Astronomy, University of California Irvine, Irvine, CA USA; 216 INFN Gruppo Collegato di Udine, Sezione di Trieste, Udine, Italy; 217 ICTP, Trieste, Italy; 218 Dipartimento di Chimica Fisica e Ambiente, Università di Udine, Udine, Italy; 219 Department of Physics and Astronomy, University of Uppsala, Uppsala, Sweden; 220 Department of Physics, University of Illinois, Urbana, IL USA; 221 Instituto de Fisica Corpuscular (IFIC) and Departamento de Fisica Atomica, Molecular y Nuclear and Departamento de Ingeniería Electrónica and Instituto de Microelectrónica de Barcelona (IMB-CNM), University of Valencia and CSIC, Valencia, Spain; 222 Department of Physics, University of British Columbia, Vancouver, BC Canada; 223 Department of Physics and Astronomy, University of Victoria, Victoria, BC Canada; 224 Department of Physics, University of Warwick, Coventry, UK; 225 Waseda University, Tokyo, Japan; 226 Department of Particle Physics, The Weizmann Institute of Science, Rehovot, Israel; 227 Department of Physics, University of Wisconsin, Madison, WI USA; 228 Fakultät für Physik und Astronomie, Julius-Maximilians-Universität, Würzburg, Germany; 229 Fakultät für Mathematik und Naturwissenschaften, Fachgruppe Physik, Bergische Universität Wuppertal, Wuppertal, Germany; 230 Department of Physics, Yale University, New Haven, CT USA; 231 Yerevan Physics Institute, Yerevan, Armenia; 232 Centre de Calcul de l’Institut National de Physique Nucléaire et de Physique des Particules (IN2P3), Villeurbanne, France; 233 CERN, 1211 Geneva 23, Switzerland

## Abstract

This paper reports a search for triboson $W^{\pm }W^{\pm }W^{\mp }$ production in two decay channels (${W^{\pm }W^{\pm }W^{\mp } \rightarrow \ell ^\pm \nu \ell ^\pm \nu \ell ^\mp \nu }$ and ${W^{\pm }W^{\pm }W^{\mp } \rightarrow \ell ^\pm \nu \ell ^\pm \nu jj}$ with $\ell =e, \mu$) in proton-proton collision data corresponding to an integrated luminosity of 20.3 $\mathrm{fb}^\mathrm{-1}$ at a centre-of-mass energy of 8 $\text {TeV}$ with the ATLAS detector at the Large Hadron Collider. Events with exactly three charged leptons, or two leptons with the same electric charge in association with two jets, are selected. The total number of events observed in data is consistent with the Standard Model (SM) predictions. The observed 95% confidence level upper limit on the SM $W^{\pm }W^{\pm }W^{\mp }$ production cross section is found to be 730 fb with an expected limit of 560 fb in the absence of SM $W^{\pm }W^{\pm }W^{\mp }$ production. Limits are also set on *WWWW* anomalous quartic gauge couplings.

## Introduction

The triple gauge couplings (TGCs) and quartic gauge couplings (QGCs) that describe the strengths of the triple and quartic gauge boson self-interactions are completely determined by the non-Abelian nature of the electroweak SU(2)$_{\text {L}} \times$ U(1)$_{\text {Y}}$ gauge structure in the Standard Model (SM). These interactions contribute directly to diboson and triboson production at colliders. Studies of triboson production can test these interactions and any possible observed deviation from the theoretical prediction would provide hints of new physics at a higher energy scale. Compared with TGCs, QGCs are usually harder to study due to the, in general, smaller production cross sections of the relevant processes.

In the SM, charged QGC interactions (*WWWW*, *WWZZ*, $WWZ\gamma$ and $WW\gamma \gamma$) are allowed whereas neutral QGC interactions (*ZZZZ*, $ZZZ\gamma$, $ZZ\gamma \gamma$, $Z\gamma \gamma \gamma$ and $\gamma \gamma \gamma \gamma$) are forbidden. Searches have been performed by the LEP experiments for $WW\gamma \gamma$, $WWZ\gamma$, and $ZZ\gamma \gamma$ QGCs [1–6], by the Tevatron experiments for $WW\gamma \gamma$ [7], and by the LHC experiments for $WW\gamma \gamma$, $WWZ\gamma$, *WWZZ*, $ZZ\gamma \gamma$, $Z\gamma \gamma \gamma$, and *WWWW* QGCs [8–17].

Previous studies of *WWWW* QGC interactions [8, 16] used $W^\pm W^\pm$ vector-boson scattering events, whereas this paper presents the first search for *WWWW* QGC interactions via triboson $W^{\pm }W^{\pm }W^{\mp }$ production and sets the first limit on the total SM $W^{\pm }W^{\pm }W^{\mp }$ production cross-section using proton-proton (*pp*) collision data collected with the ATLAS detector and corresponding to an integrated luminosity of 20.3 $\mathrm{fb}^\mathrm{-1}$ [18] at a centre-of-mass energy of 8 $\text {TeV}$. Two decay channels, $W^{\pm }W^{\pm }W^{\mp } \rightarrow \ell ^\pm \nu \ell ^\pm \nu \ell ^\mp \nu$ and $W^{\pm }W^{\pm }W^{\mp } \rightarrow \ell ^\pm \nu \ell ^\pm \nu jj$ , with $\ell =e$ or $\mu$, are considered and are hereafter referred to simply as $\ell \nu \ell \nu \ell \nu$ and $\ell \nu \ell \nu jj$ channels, respectively.

## The ATLAS detector

The ATLAS detector [19] is composed of an inner tracking detector (ID) surrounded by a thin superconducting solenoid providing a 2 T axial magnetic field, electromagnetic and hadronic calorimeters, and a muon spectrometer (MS). The ID consists of three subsystems: the pixel and silicon microstrip detectors that cover $|\eta |<2.5$ in pseudorapidity,^1^ and the outer transition radiation tracker that has an acceptance range of $|\eta |<2.0$. The finely-segmented electromagnetic calorimeter is composed of lead absorbers with liquid argon (LAr) as the active material, spanning $|\eta |<3.2$. In the region $|\eta |<1.8$, a pre-sampler detector using a thin layer of LAr is used to correct for the energy loss by electrons and photons upstream of the calorimeter. The hadronic tile calorimeter ($|\eta |<1.7$) consists of steel absorbers and scintillating tiles and is located directly outside the envelope of the barrel electromagnetic calorimeter. The endcap hadronic calorimeters use LAr as active material, with copper as absorber material, while the forward calorimeters use LAr as active material, with copper absorber for the first layer, dedicated to electromagnetic measurements, and tungsten for other layers, dedicated to hadronic measurements. The MS is composed of three large superconducting air-core toroidal magnets, a system of three stations of tracking chambers in the range $|\eta |<2.7$, and a muon trigger system in the range $|\eta |< 2.4$. The precision muon momentum measurement is performed by monitored drift tubes everywhere except in the innermost layer for the range $|\eta | > 2.0$ where cathode strip chambers are used instead. The muon trigger system is composed of resistive plate chambers in the barrel region ($|\eta | < 1.05$) and thin gap chambers in the endcap region ($1.05< |\eta | < 2.4$).

The ATLAS trigger system has three distinct levels referred to as L1, L2, and the event filter. Each trigger level refines the decisions made at the previous level. The L1 trigger is implemented in hardware and uses a subset of detector information to reduce the event rate to a design value of at most 75 kHz. The L2 and event filter are software-based trigger levels and together reduce the event rate to about 400 Hz.

Events used were selected by single-lepton triggers with a transverse momentum, $p_{\text {T}}$ , threshold of 24 $\text {GeV}$ for both muons and electrons, along with an isolation requirement. The single-lepton triggers are complemented with triggers having a higher $p_{\text {T}}$ threshold (60 $\text {GeV}$ for electrons and 36 $\text {GeV}$ for muons) and no isolation requirement in order to increase the acceptance at high $p_{\text {T}}$.

## Object reconstruction and event selection

Each event is required to have at least one primary vertex reconstructed from at least three tracks with $p_{\text {T}} >400$ $\text {MeV}$. If there are multiple primary vertices reconstructed in the event due to additional *pp* interactions (pile-up) in the same or a neighbouring bunch crossing, the vertex with the highest $\sum p_{\text {T}} ^2$, calculated using all associated tracks, is taken as the primary collision vertex. The mean number of interactions per bunch crossing in this data set is 20.7.

Electron candidates [20] are required to have $p_{\text {T}} >20$ $\text {GeV}$ and $|\eta | < 2.47$. Candidates within the transition region between the barrel and endcap calorimeters ($1.37< |\eta | < 1.52$) are rejected. In addition, they must satisfy the *tight* quality definition described in Ref. [21]. Muon candidates are reconstructed by combining tracks in the ID with tracks in the MS and have $p_{\text {T}} >20$ $\text {GeV}$ and $|\eta |<2.5$. The ID tracks associated with these muons must pass a number of quality requirements [22].

To ensure that lepton candidates originate from the primary vertex, a requirement is placed on the longitudinal impact parameter, $z_0$, multiplied by the sine of the track polar angle, $\theta$, such that the absolute value is smaller than 0.5 mm ($|z_0 \times \sin \theta |<0.5$ mm). A requirement is also placed on the transverse impact parameter, $d_0$, divided by its resolution ($\sigma _{d_0}$), such that $|d_0/\sigma _{d_0}|<3$. To suppress the contribution from hadronic jets which are misidentified as leptons, signal leptons are required to be isolated in both the ID and the calorimeter. The calorimeter isolation is defined as $E_{\text {T}} ^{\mathrm {Cone}X}/E_{\text {T}}$ whereas the ID isolation is defined as $p_{\text {T}} ^{\mathrm {Cone}X}/p_{\text {T}}$, where $E_{\text {T}} ^{\mathrm {Cone}X}$ ($p_{\text {T}} ^{\mathrm {Cone}X}$) is the transverse energy (momentum) deposited in the calorimeter (the scalar sum of the $p_{\text {T}}$ of tracks with $p_{\text {T}} >1$ $\text {GeV}$) within a cone of size $\Delta R=\sqrt{(\Delta \eta )^2 + (\Delta \phi )^2}=X$ around the lepton. The transverse momentum from the lepton itself is excluded in the calculations of $E_{\text {T}} ^{\mathrm {Cone}X}$ and $p_{\text {T}} ^{\mathrm {Cone}X}$. Different lepton isolation criteria are applied in the two channels to maximize the signal efficiency while suppressing the backgrounds. In the $\ell \nu \ell \nu \ell \nu$ channel, $E_{\text {T}} ^{\mathrm {Cone}0.2}/E_{\text {T}} <0.1$ and $p_{\text {T}} ^{\mathrm {Cone}0.2}/p_{\text {T}} <0.04$ are required for both the electrons and muons; in the $\ell \nu \ell \nu jj$ channel, $E_{\text {T}} ^{\mathrm {Cone}0.3}/E_{\text {T}} <0.14$ and $p_{\text {T}} ^{\mathrm {Cone}0.3}/p_{\text {T}} <0.06$ are required for electrons whereas $E_{\text {T}} ^{\mathrm {Cone}0.3}/E_{\text {T}} <0.07$ and $p_{\text {T}} ^{\mathrm {Cone}0.3}/p_{\text {T}} <0.07$ are required for muons.

Jets are reconstructed from clusters of energy in the calorimeter using the anti-$k_t$ algorithm [23] with radius parameter $R = 0.4$. Jet energies are calibrated using energy- and $\eta$-dependent correction factors derived using Monte Carlo (MC) simulation and validated by studies of collision data [24]. For jets with $p_{\text {T}} <50$ $\text {GeV}$ and $|\eta |<2.4$, at least 50% of the summed scalar $p_{\text {T}}$  of the tracks within a cone of size $\Delta R=0.4$ around the jet axis must originate from the primary vertex. This requirement reduces the number of jet candidates originating from pile-up vertices. Jets containing *b*-hadrons (“*b*-jets”) with $|\eta | < 2.5$ and $p_{\text {T}} >25~\text {GeV}$ are identified using the impact parameter significance of tracks in the jet and secondary vertices reconstructed from these tracks [25, 26]. In the $\ell \nu \ell \nu \ell \nu$ and $\ell \nu \ell \nu jj$ channels, the efficiency of the *b*-tagging algorithm used is 85 and 70%, respectively.

The measurement of the two-dimensional missing transverse momentum vector, $\vec {p}_{\text {T}}^{\text {~miss}}$, is based on the measurement of all topological clusters in the calorimeter and muon tracks reconstructed in the ID and MS [27]. Calorimeter cells associated with reconstructed objects, such as electrons, photons, hadronically decaying $\tau$ leptons, and jets, are calibrated at their own energy scale, whereas calorimeter cells not associated with any object are calibrated at the electromagnetic energy scale and taken into account as a so-called “soft term” in the calculation of $\vec {p}_{\text {T}}^{\text {~miss}}$. The magnitude of the missing transverse momentum vector is referred to as the missing transverse energy, $E_{\text {T}}^{\text {miss}} = |\vec {p}_{\text {T}}^{\text {~miss}} |$.

The experimental signature of the $\ell \nu \ell \nu \ell \nu$ channel is the presence of three charged leptons and $E_{\text {T}}^{\text {miss}}$ . The signature of the $\ell \nu \ell \nu jj$ channel is the presence of two same-charge leptons, $E_{\text {T}}^{\text {miss}}$, and two jets with an invariant mass close to 80 $\text {GeV}$. The selection requirements used to define the signal regions described in the following are obtained from a multi-dimensional optimization to maximize the sensitivity to the $W^{\pm }W^{\pm }W^{\mp }$ process and to reduce the contributions from SM background processes.

To select $\ell \nu \ell \nu \ell \nu$ candidates, events are required to have exactly three charged leptons with $p_{\text {T}} >20$ $\text {GeV}$, at most one jet with $p_{\text {T}} >25$ $\text {GeV}$ and $|\eta |<4.5$, and no identified *b*-jets. In addition, the absolute value of the azimuthal angle between the trilepton system and the $\vec {p}_{\text {T}}^{\text {~miss}}$, $|\phi ^{3\ell } - \phi ^{\vec {p}_{\text {T}}^{\text {~miss}}}|$, is required to be above 2.5. Eight different final states with equal production probability are considered based on the flavour and the charge of the leptons, namely $e^\pm e^\pm e^\mp$, $e^{\pm }e^{\mp }\mu ^{\pm }$, $e^{\pm }e^{\mp }\mu ^{\mp }$, $e^\pm e^\pm \mu ^\mp$, $\mu ^{\pm }\mu ^{\mp }e^{\pm }$, $\mu ^{\pm }\mu ^{\mp }e^{\mp }$, $\mu ^\pm \mu ^\pm e^\mp$, and $\mu ^\pm \mu ^\pm \mu ^\mp$. Three separate signal regions are defined based on the number of same-flavour opposite-sign (SFOS) lepton pairs in the event: 0 SFOS ($e^\pm e^\pm \mu ^\mp$ and $\mu ^\pm \mu ^\pm e^\mp$), 1 SFOS ($e^{\pm }e^{\mp }\mu ^{\pm }$, $e^{\pm }e^{\mp }\mu ^{\mp }$, $\mu ^{\pm }\mu ^{\mp }e^{\pm }$, and $\mu ^{\pm }\mu ^{\mp }e^{\mp }$), and 2 SFOS ($e^\pm e^\pm e^\mp$ and $\mu ^\pm \mu ^\pm \mu ^\mp$). In the 0-SFOS case, the invariant mass of the same-flavour lepton pair, $m_{\ell \ell }$, is required to be greater than 20 $\text {GeV}$. If there are at least two electrons in the event, the di-electron invariant mass, $m_{ee}$, is required to have $|m_{ee} - m_Z|>15$ $\text {GeV}$, where $m_Z$ is the pole mass of the *Z* boson [28]. No requirement is applied on the $E_{\text {T}}^{\text {miss}}$ variable, as it was found to not discriminate between signal and backgrounds. In the 1-SFOS case, the SFOS dilepton invariant mass, $m_\mathrm{SFOS}$, is required to be outside of the region $m_Z-35~\text {GeV}{}<m_{\text {SFOS}}<m_Z+20$ $\text {GeV}$. In addition, events are required to satisfy $E_{\text {T}}^{\text {miss}} >45$ $\text {GeV}$. Finally, in the 2-SFOS case, the SFOS dilepton invariant masses are required to have $|m_\mathrm{SFOS}-m_Z|>20$ $\text {GeV}$ while the $E_{\text {T}}^{\text {miss}}$  must be greater than 55 $\text {GeV}$. The selection criteria for $m_\mathrm{SFOS}$ and $E_{\text {T}}^{\text {miss}}$ are mainly used to reduce the contributions from the $Z+$jets and $WZ+$jets processes. Table 1 shows the kinematic selection criteria used for the $\ell \nu \ell \nu \ell \nu$ channel.

**Table 1:** Selection criteria for the $\ell \nu \ell \nu \ell \nu$ channel, split based on the number of SFOS lepton pairs: 0 SFOS, 1 SFOS, and 2 SFOS

$\ell \nu \ell \nu \ell \nu$	0 SFOS	1 SFOS	2 SFOS
Preselection	Exactly three charged leptons with $p_{\text {T}} >20$ $\text {GeV}$		
$E_{\text {T}}^{\text {miss}}$	–	$E_{\text {T}}^{\text {miss}} >45$ $\text {GeV}$	$E_{\text {T}}^{\text {miss}} >55$ $\text {GeV}$
Same-flavour dilepton mass	$m_{\ell \ell }>20$ $\text {GeV}$	–	
Angle between trilepton and $\vec {p}_{\text {T}}^{\text {~miss}}$	$|\phi ^{3\ell } - \phi ^{\vec {p}_{\text {T}}^{\text {~miss}}}|>2.5$		
*Z* boson veto	$|m_{ee}-m_Z|>15$ $\text {GeV}$	$m_Z - m_{\text {SFOS}} > 35$ $\text {GeV}$	$|m_{\text {SFOS}}-m_Z|>20$ $\text {GeV}$
		or	
		$m_{\text {SFOS}} - m_Z > 20$ $\text {GeV}$	
Jet veto	At most one jet with $p_{\text {T}} >25$ $\text {GeV}$ and $|\eta |<4.5$		
*b*-jet veto	No identified $b-$jets with $p_{\text {T}} >25$ $\text {GeV}$ and $|\eta |<2.5$		

To select $\ell \nu \ell \nu jj$ candidates, events are required to have exactly two leptons with the same electric charge, at least two jets, and no identified *b*-jets. Three different final states are considered based on the lepton flavour, namely $e^\pm e^\pm$, $e^\pm \mu ^\pm$, and $\mu ^\pm \mu ^\pm$. The lepton $p_{\text {T}}$  ($E_{\text {T}}^{\text {miss}}$) threshold is set to 30 (55) $\text {GeV}$ to reduce the SM background contributions, though the $E_{\text {T}}^{\text {miss}}$ criterion is not applied for the $\mu ^\pm \mu ^\pm$ final state due to the smaller $Z+$jets background expected in this channel. The leading (sub-leading) $p_{\text {T}}$ jet must have $p_{\text {T}} >30$ (20) $\text {GeV}$ and $|\eta |<2.5$. The two jets are required to have $65~\text {GeV}{}<m_{jj}<105$ $\text {GeV}$ and $|\Delta \eta _{jj}|<1.5$ in order to distinguish the signal from the $W^\pm W^\pm$ backgrounds, where $m_{jj}$ is the dijet invariant mass and $\Delta \eta _{jj}$ is the pseudorapidity separation between the two jets. The dilepton system is required to have $m_{\ell \ell }>40$ $\text {GeV}$ and in the case of the $e^\pm e^\pm$ final state, $m_{ee}$ must have $m_{ee}<80$ $\text {GeV}$ or $m_{ee}>100$ $\text {GeV}$ in order to suppress events with two opposite-sign prompt leptons where the charge of one of the electrons is misidentified. To reduce the contributions from $WZ+$jets and $ZZ+$jets production, events are removed if they contain additional leptons reconstructed with $p_{\text {T}} >6$ $\text {GeV}$ passing looser identification quality requirements, with a *medium* identification requirement for electrons as defined in Ref. [21] and the minimum identification required for muon reconstruction. Table 2 shows the kinematic selection criteria used for the $\ell \nu \ell \nu jj$ channel.

**Table 2:** Selection criteria for the $\ell \nu \ell \nu jj$ channel, split based on the lepton flavour: $e^\pm e^\pm$ , $e^\pm \mu ^\pm$ , and $\mu ^\pm \mu ^\pm$

$\ell \nu \ell \nu jj$	$e^\pm e^\pm$	$e^\pm \mu ^\pm$	$\mu ^\pm \mu ^\pm$
Lepton	Exactly two same-charge leptons with $p_{\text {T}} >30$ $\text {GeV}$		
Jets	At least two jets with $p_{\text {T}} (1)>30$ $\text {GeV}$, $p_{\text {T}} (2)>20$ $\text {GeV}$ and $|\eta |<2.5$		
$m_{\ell \ell }$	$m_{\ell \ell }>40$ $\text {GeV}$		
$E_{\text {T}}^{\text {miss}}$	$E_{\text {T}}^{\text {miss}} >55$ $\text {GeV}$		–
$m_{jj}$	$65~\text {GeV}<m_{jj}<105$ $\text {GeV}$		
$\Delta \eta _{jj}$	$|\Delta \eta _{jj}|<1.5$		
*Z* boson veto	$m_{ee}<80$ $\text {GeV}$ or $m_{ee}>100$ $\text {GeV}$	–	
Third-lepton veto	No third lepton with $p_{\text {T}} >6$ $\text {GeV}$ and $|\eta |<2.5$ passing looser identification requirements		
*b*-jet veto	No identified *b*-jets with $p_{\text {T}} >25$ $\text {GeV}$ and $|\eta |<2.5$		

## Signal fiducial cross sections

**Fig. 1:**
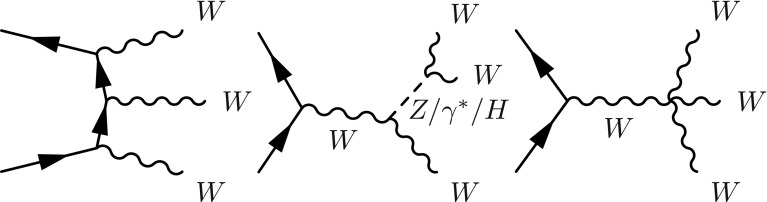
Feynman graphs contributing at LO to $W^{\pm }W^{\pm }W^{\mp }$ production

At leading order (LO), the production of three *W* bosons can take place through radiation from a fermion, from an associated *W* and $Z/\gamma ^*/H$ production with the intermediate $Z/\gamma ^*/H$ boson decaying to two opposite-sign *W* bosons, or from a *WWWW* QGC vertex. Representative Feynman graphs for each of these production processes are shown in Fig. 1. Calculations are available including corrections at next-to-leading order (NLO) in QCD with all spin correlations involved in the vector-boson decays, the effects due to intermediate Higgs boson exchange, and off-shell contributions correctly taken into account [29]. Electroweak NLO corrections have been calculated recently [30]. However, they are not considered in this analysis.

In order to determine $W^{\pm }W^{\pm }W^{\mp }$ production cross sections, events are generated at NLO in QCD using MadGraph5_aMC@NLO  [31] including on-shell diagrams as well as Higgs associated diagrams. The CT10 NLO parton distribution function (PDF) [32] is used. Subsequent decays of unstable particles and parton showers are handled by pythia8 [33]. Fiducial cross sections are calculated using the generator-level lepton, jet, and $E_{\text {T}}^{\text {miss}}$ definitions as described in Ref. [34]. Generator-level prompt leptons (those not originating from hadron and $\tau$ lepton decays) are dressed with prompt photons within a cone of size $\Delta R = 0.1$. Generator-level jets are reconstructed by applying the anti-$k_t$ algorithm with radius parameter $R=0.4$ on all final-state particles after parton showering and hadronisation. The $E_{\text {T}}^{\text {miss}}$ variable is calculated using all generator-level neutrinos. The same kinematic selection criteria as listed in Tables 1 and 2 are applied on these objects, with the exception of the *b*-jet veto requirements in the $\ell \nu \ell \nu \ell \nu$ channel and the lepton quality requirements. To take into account the effect of the lepton isolation in the fiducial region, any lepton pairs must satisfy $\Delta R(\ell, \ell)>0.1$, and in the $\ell \nu \ell \nu jj$ channel any lepton-jet pairs must satisfy $\Delta R(j, \ell)>0.3$. Electrons or muons from $\tau$ decays are not included.

The fiducial cross section is predicted to be $309 \pm 7~(\mathrm{stat.}) \pm {}15~(\mathrm{PDF}) \pm 8~(\mathrm{scale})$ ab in the $\ell \nu \ell \nu \ell \nu$ channel and $286 \pm 6~(\mathrm{stat.}) \pm {}15~(\mathrm{PDF}) \pm 10~(\mathrm{scale})$ ab in the $\ell \nu \ell \nu jj$ channel. Uncertainties due to the PDFs are computed using an envelope of the CT10, NNPDF3.0 [35], and MSTW2008 [36] NLO PDF 68 or 90% (for CT10) confidence level (CL) uncertainties, following the recommendation of Ref. [37]. The renormalization and factorization scales are set to the invariant mass of the *WWW* system. Scale uncertainties are estimated by varying the two scales independently up and down by a factor of two and taking the largest variation from the nominal cross-section values.

In order to combine the measurements from the two decay channels, a common phase space is defined where each *W* boson can decay either leptonically (including $\tau$ leptons) or hadronically, $pp\rightarrow W^\pm W^\pm W^\mp$ $+~X$, with no kinematic requirements placed on the final-state leptons but with jets restricted to have $p_{\text {T}} > 10$ $\text {GeV}$. The extrapolation factor from the fiducial phase space to the total phase space is large, but it is mainly due to the well-known *W* boson decay branching ratios. The total cross section in this common phase space is $241.5 \pm 0.1$ (stat.) $\pm 10.3$ (PDF) $\pm 6.3$ (scale) fb.

In order to determine the detector reconstruction effects on the signal selection, $W^{\pm }W^{\pm }W^{\mp }$ signal samples are generated with vbfnlo [29, 38–40] at LO. The parton shower and hadronisation are performed by pythia8. The fiducial cross sections are seen to be consistent between vbfnlo and MadGraph5_aMC@NLO when computed at the same order. The vbfnlo LO fiducial cross sections are normalized to the NLO fiducial cross section predicted by MadGraph5_aMC@NLO for the signal yield calculations. These events are processed through the full ATLAS detector simulation [41] based on Geant 4 [42]. To simulate the effect of multiple *pp* interactions occurring during the same or a neighbouring bunch crossing, minimum-bias interactions are generated and overlaid on the hard-scattering process. These events are then processed through the same object reconstruction and identification algorithms as used on data. MC events are reweighted so that the pile-up conditions in the simulation match the data. Additional corrections are made to the simulated samples to account for small differences between the simulation and the data for the object identification and reconstruction efficiencies, the trigger efficiencies, and the energy and momentum scales and resolutions. While excluded in the fiducial cross-section definition, the contribution from events with $W\rightarrow \tau \nu \rightarrow \ell \nu \nu \nu$ decays are counted as signal in the vbfnlo signal sample used in the final event selection. These events contribute up to 20% of the predicted signal yield. This approach is used to ease comparisons of the obtained cross-section limits with alternative cross-section predictions that may not simulate tau decays.

## Backgrounds

### Background estimation

The SM processes that mimic the $W^{\pm }W^{\pm }W^{\mp }$ signal signature can be grouped into five categories:

- The $WZ/\gamma ^*+$jets process that produces three prompt leptons or two prompt leptons with the same electric charge (referred to as “*WZ* background”);
- The $W\gamma +$jets or $Z\gamma +$jets processes where the photon is misreconstructed as a lepton (referred to as “$V\gamma$ background”, where $V=W, Z$);
- Processes other than $WZ/\gamma ^*+$jets that produce three prompt leptons or two prompt leptons with the same electric charge (referred to as “other prompt background”);
- Processes that produce two or three prompt charged leptons, but the charge of one lepton is misidentified (referred to as “charge-flip background”);
- Processes that have one or two non-prompt leptons originating either from misidentified jets or from hadronic decays (referred to as “fake-lepton background”).

The dominant irreducible background originates from the $WZ(\rightarrow \ell ^\pm \nu \ell ^\pm \ell ^\mp )+$jets process and is estimated using simulated events. In the $\ell \nu \ell \nu \ell \nu$ channel these events are generated with Powheg -BOX [43–46] and hadronised with pythia8 and in the $\ell \nu \ell \nu jj$ channel they are generated with Sherpa  [47]. In the $\ell \nu \ell \nu \ell \nu$ channel, the inclusive $WZ+$jets cross section is normalized using a scale factor ($1.08\pm 0.10$) derived from a *WZ*-enriched region in data. This region is obtained by requiring exactly one SFOS lepton pair with $|m_\mathrm{SFOS}-m_Z|<15~\text {GeV}$. In the $\ell \nu \ell \nu jj$ channel, the cross section is normalized to the NLO calculation in QCD from vbfnlo [48] in the specified fiducial phase space with a normalization factor of $1.04\pm 0.09$.

The $V\gamma$ background contributes when the photon is misidentified as an electron. In the $\ell \nu \ell \nu \ell \nu$ channel, this originates primarily from the $Z\gamma$ process and its contribution is estimated using events generated with Sherpa . In the $\ell \nu \ell \nu jj$ channel, this comes primarily from electroweak and strong production of $W\gamma jj$ events. Strong production of $W\gamma jj$ [49] is estimated using Alpgen  [50] interfaced to Herwig  [51] and Jimmy  [52] for simulation of the parton shower, fragmentation, hadronisation and the underlying event. The electroweak production of $W\gamma jj$ [53] is modelled using Sherpa .

Other SM processes that produce multiple prompt leptons include *ZZ*, $t\bar{t}V$, *ZWW*, *ZZZ*, $W^\pm W^\pm jj$ production, and double parton scattering processes. The production of *ZZ* is modelled with Powheg -BOX [46] and hadronised with pythia8 in the $\ell \nu \ell \nu \ell \nu$ channel and is modelled with Sherpa in the $\ell \nu \ell \nu jj$ channel. The $t\bar{t}V$ [54], *ZWW* [55], and *ZZZ* [55] processes are modelled using MadGraph5_aMC@NLO together with pythia8 for both channels. The non-resonant $W^\pm W^\pm jj$ background [56] is only important for the $\ell \nu \ell \nu jj$ channel and its contribution is estimated using Sherpa. Contributions from double parton scattering processes are found to be negligible in both channels.

The charge-flip background originates from processes where the charge of at least one prompt lepton is misidentified. This occurs primarily when a lepton from a hard bremsstrahlung photon conversion is recorded instead of the signal lepton. It mainly contributes to the 0-SFOS signal region in the $\ell \nu \ell \nu \ell \nu$ channel and the $e^\pm e^\pm$ / $e^\pm \mu ^\pm$ signal regions in the $\ell \nu \ell \nu jj$ channel. The electron charge misidentification rate is measured using $Z \rightarrow e^+e^-$ events. In the $\ell \nu \ell \nu \ell \nu$ channel, the charge-flip background is estimated by using these rates to re-weight the MC estimate of *WZ* and *ZZ* events based on the probability for opposite-sign events of this kind to migrate into the 0-SFOS category. In the $\ell \nu \ell \nu jj$ channel, the background is estimated by applying these rates on data events satisfying all signal selection criteria except the two leptons are required to have opposite-sign.

Contributions from fake-lepton backgrounds are estimated in data, using different approaches in the two channels. In the $\ell \nu \ell \nu \ell \nu$ channel, the probabilities of prompt leptons or non-prompt leptons to satisfy the signal lepton criteria are computed using a tag-and-probe method whereby a well-reconstructed “tag” lepton is used to identify the event and a second “probe” lepton is used to study the probabilities without bias. A tag lepton must satisfy the signal lepton requirements while a looser lepton selection criterion is defined for probe leptons with the lepton isolation requirements removed and the electron quality requirement loosened to *medium* as defined in Ref. [21]. The probability for a prompt lepton to satisfy the signal lepton criteria is estimated using $Z \rightarrow \ell ^+ \ell ^-$ events with the tag-and-probe lepton pair required to have the same-flavour, opposite-sign and an invariant mass within 10 $\text {GeV}$ of the pole mass of the *Z* boson. The probability for a non-prompt lepton from hadronic activity to satisfy the signal lepton requirement is estimated using the tag-and-probe method in a $W+$jets-enriched region with $E_{\text {T}}^{\text {miss}} > 10$ $\text {GeV}$, the tag lepton is a muon with $p_{\text {T}} > 40$ $\text {GeV}$, and the tag and probe leptons have the same electric charge. The probabilities are calculated separately for electrons and muons. A loosely identified set of data is also selected by requiring at least three loose leptons as defined above. This set of data, along with these probabilities are then used to estimate the background in the signal region with the matrix method [57].

In the $\ell \nu \ell \nu jj$ channel, events that contain one signal lepton and one “lepton-like” jet are selected. A “lepton-like” jet satisfies all signal lepton selection criteria except that the isolation requirements are $0.14<E_{\text {T}} ^{\mathrm {Cone}0.3}/p_{\text {T}} <2$ and $0.06<p_{\text {T}} ^{\mathrm {Cone}0.3}/p_{\text {T}} <2$ for electrons, and $0.07<E_{\text {T}} ^{\mathrm {Cone}0.3}/p_{\text {T}} <2$ and $0.07<p_{\text {T}} ^{\mathrm {Cone}0.3}/p_{\text {T}} <2$ for muons. In addition, the $|d_0/\sigma _{d_0}|$ and $|z_0 \times \sin \theta |$ selection criteria are loosened to 10 mm and 5 mm, respectively. These events are dominated by non-prompt leptons and are scaled by a fake factor to estimate the non-prompt background. The fake factor is the ratio of the number of jets satisfying the signal lepton identification criteria to the number of jets satisfying the “lepton-like” jet criteria. It is measured as a function of the jet $p_{\text {T}}$ and $\eta$ from a dijet-enriched sample selected by requiring a lepton back-to-back with a jet ($\Delta \phi _{j\ell } > 2.8$) and $E_{\text {T}}^{\text {miss}} < 40$ $\text {GeV}$.

### Validation of background estimates

The background predictions are tested in several validation regions (VRs). These VRs are defined to be close to the signal region with a few selection criteria removed or inverted. They generally have dominant contributions from one or two background sources and a negligible contribution from the signal process. The signal and background predictions are compared to data for each VR in Table 3.

**Fig. 2:**
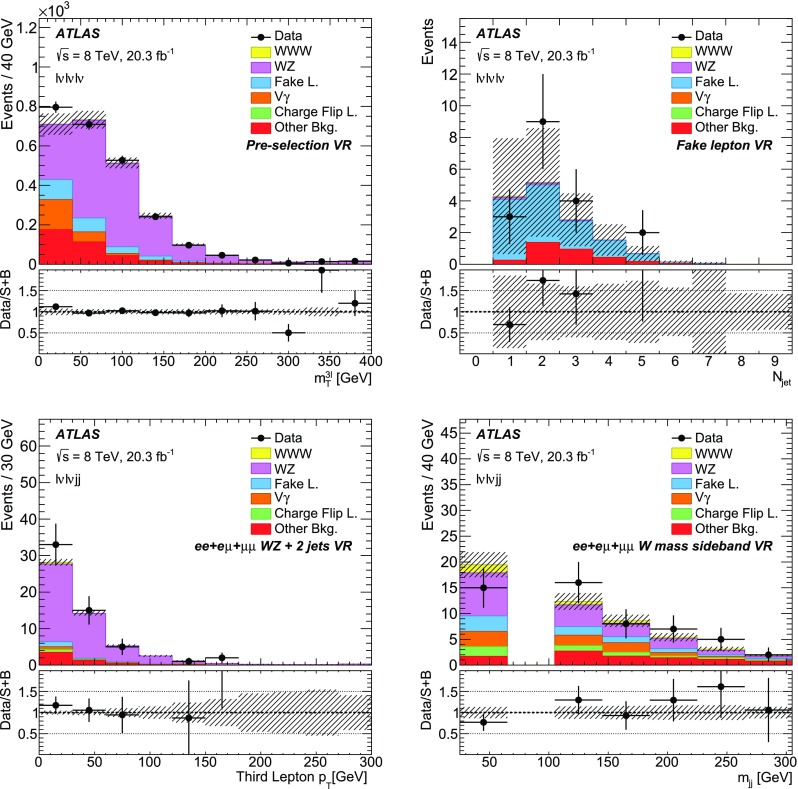
Distributions in four different VRs, two corresponding to the $\ell \nu \ell \nu \ell \nu$ channel (top) and two to the $\ell \nu \ell \nu jj$ channel (*bottom*). For the $\ell \nu \ell \nu \ell \nu$ channel the $m_T^{3\ell }$ distribution in the preselection region (*top left*) and the jet multiplicity distribution in the fake-lepton region (*top right*) are shown. For the $\ell \nu \ell \nu jj$ channel the third-lepton $p_{\text {T}}$ in the $WZ+2$-jets region (*bottom left*) and the $m_{jj}$ distribution in the *W* mass sideband region (*bottom right*) are shown. The “other backgrounds” contain prompt leptons and are estimated from MC. The *hashed band* represents total uncertainties on the signal-plus-background prediction. The highest bin also includes events falling out of the range shown

In the $\ell \nu \ell \nu \ell \nu$ channel, three VRs are considered. The first VR, called the pre-selection region, tests the modelling of the *WZ*+jets background by requiring exactly three signal leptons. The distribution of the trilepton transverse mass, $m_{T}^{3\ell } = \sqrt{2 p_{\text {T}} ^{3\ell }E_{\text {T}}^{\text {miss}} \Big (1-\cos (\phi ^{3\ell } - \phi ^{\vec {p}_{\text {T}}^{\text {~miss}}})\Big )}$ where $p_{\text {T}} ^{3\ell }$ is the $p_{\text {T}}$ of the trilepton system, is shown at the top left of Fig. 2. This VR includes the three signal regions (0, 1, and 2 SFOS), but the effect of the signal is considered negligible at this stage of the selection, as shown in Table 3. The *WZ*+jets purity is estimated to be around $70\%$ in this region. The second region, called the fake-lepton region, tests the modelling of the fake-lepton background by requiring exactly three signal leptons with no SFOS lepton pairs and at least one $b-$jet. The distribution of the jet multiplicity, $N_{\text {jet}}$, is shown at the top right of Fig. 2. The purity of the fake-lepton background is estimated to be around $80\%$ in this region. The third region, called $Z\gamma$ region, tests the modelling of the $Z\gamma$ background by requiring the presence of only $\mu ^+\mu ^- e^\pm$ events where the trilepton invariant mass is close to the *Z* resonance peak. This restricts the main contributions to originate from the $Z\gamma \rightarrow \mu ^+ \mu ^- \gamma$ and $Z \rightarrow \mu ^+ \mu ^- \rightarrow \mu ^+ \mu ^- \gamma$ processes. The $Z\gamma$ purity is estimated to be around $70\%$ in this region. The data are seen to be well described by the background in all three VRs.

In the $\ell \nu \ell \nu jj$ channel, five VRs are considered. The modelling of the charge-flip background is tested using $e^\pm e^\pm$ events with $80~\text {GeV}{}<m_{\ell \ell }<100~\text {GeV}$. The purity of the charge-flip background is estimated to be around $80\%$ in this region. The modelling of the $WZ+$jets background is checked in a $WZ+2$-jets region requiring the presence of an additional lepton. The $p_{\text {T}}$ of this third lepton is shown at the bottom left of Fig. 2. The purity of the *WZ*+jets is estimated to be around $60\%$ in this region. The modelling of backgrounds from non-prompt leptons is tested in a $b-$tagged region that requires at least one $b-$jet. The purity of the non-prompt lepton background is estimated to be around $80\%$ in this region. The $m_{jj}$ modelling is checked by examining events with masses $m_{jj}$ in the regions $m_{jj}<65~\text {GeV}$ or $m_{jj}>105~\text {GeV}$. The distribution of $m_{jj}$ in this region is shown at the bottom right of Fig. 2. Finally, conversion and prompt backgrounds are tested in a region with at most one jet, called the $\le 1$ jet region. The purity of the conversion and prompt backgrounds is estimated to be around $70\%$ in this region. As for the $\ell \nu \ell \nu \ell \nu$ channel, good agreement is observed between the data and the prediction in all five VRs.

**Table 3:** Expected numbers of signal and background events in the VRs compared to the numbers of events observed in data. The first uncertainty is statistical and the second is systematic

Validation Region	Signal	Background	Observed
$$\ell \nu \ell \nu \ell \nu $$			
Preselection	$9.78 \pm 0.04 \pm 0.45$	$2392 \pm 7 \pm 298$	2472
Fake-lepton	$0.15 \pm 0.01 \pm 0.02$	$15 \pm 1 \pm 10$	18
$Z\gamma$	$0.32 \pm 0.01 \pm 0.02$	$119 \pm 3 \pm 20$	119
$$\ell \nu \ell \nu jj$$			
Charge-flip	$0.98 \pm 0.04 \pm 0.06$	$21 \pm 1 \pm 2$	22
$WZ+2$-jets	$0.55 \pm 0.03 \pm 0.04$	$52 \pm 1 \pm 10$	56
*b*-tagged	$1.00 \pm 0.05 \pm 0.07$	$69 \pm 1 \pm 23$	78
*W* mass sideband	$3.35 \pm 0.08 \pm 0.43$	$48 \pm 2 \pm 6$	53
$\le$ 1jet	$1.62 \pm 0.06 \pm 0.40$	$139 \pm 3 \pm 18$	145

## Systematic uncertainties

Systematic uncertainties in the signal and background predictions arise from the measurement of the integrated luminosity, from the experimental and theoretical modelling of the signal acceptance and detection efficiency, and from the background estimation. The effect of the systematic uncertainties on the overall signal and background yields are evaluated separately for the $\ell \nu \ell \nu \ell \nu$ and $\ell \nu \ell \nu jj$ channels . The results are summarised in Table 4. The systematic uncertainties are included as nuisance parameters in the profile likelihood described in Sect. 7. Correlations of systematic uncertainties arising from common sources are maintained across signal and background processes and channels.

**Table 4:** The effect of the various systematic uncertainties on the total signal and background yields (in percent) for both channels

Source of uncertainty	$\ell \nu \ell \nu \ell \nu$		$\ell \nu \ell \nu jj$	
	Signal (%)	Background (%)	Signal (%)	Background (%)
Lepton ID, $E_{\text {T}}$/$p_{\text {T}}$ scale and resolution	1.6	1.8	2.1	3.3
$E_{\text {T}}^{\text {miss}}$ modelling	1.1	1.4	0.7	1.8
*b*-jet identification	0.3	0.3	2.2	2.2
Jet $E_{\text {T}}$ scale and resolution	2.3	2.8	21	15
Fake-lepton background	0	13	0	8
Charge-flip background	0	0.04	0	2.2
Luminosity	1.9	1.6	1.9	1.4
Pile-up estimate	1.1	0.6	0.6	1.6
Trigger efficiency	0.1	0.1	0.1	0.01
Normalization factor	3.8	8	6.0	13
Statistical	1.2	3.2	2.7	5.1

The experimental uncertainties include the uncertainties on the lepton and jet energy and momentum scales and resolutions, on the efficiencies of the lepton and jet reconstruction and identification, and on the modelling of $E_{\text {T}}^{\text {miss}}$ and *b*-jets. They are evaluated separately for both the signal and background estimations. For the expected signal yield, the major contributions in the $\ell \nu \ell \nu \ell \nu$ channel come from uncertainties in the lepton reconstruction and identification efficiencies as well as lepton energy/momentum resolution and scale modelling ($\pm 1.6\%$), $E_{\text {T}}^{\text {miss}}$ modelling ($\pm 1.1\%$), and jet energy scale and resolution ($\pm 2.3\%$). The contributions in the $\ell \nu \ell \nu jj$ channel come from uncertainties in the lepton efficiencies and energy/momentum modelling ($\pm 2.1\%$), $E_{\text {T}}^{\text {miss}}$ modelling ($\pm 0.7\%$), *b*-jet identification ($\pm 2.2\%$), and jet energy resolution and scale modelling ($\pm 21\%$). Larger systematic uncertainties due to the jet energy scale and resolution are expected in the $\ell \nu \ell \nu jj$ channel due to the dijet requirements, in particular in the dijet invariant mass. For the background yields estimated from MC simulation, the major contributions in the $\ell \nu \ell \nu \ell \nu$ channel come from uncertainties in lepton reconstruction and identification efficiencies ($\pm 1.8\%$), $E_{\text {T}}^{\text {miss}}$ modelling ($\pm 1.4\%$), and jet energy modelling ($\pm 2.8\%$). The major contributions in the $\ell \nu \ell \nu jj$ channel come from uncertainties in lepton efficiencies and energy modelling ($\pm 3.3\%$), $E_{\text {T}}^{\text {miss}}$ modelling ($\pm 1.8\%$), *b*-jet identification ($\pm 2.2\%$), and jet energy modelling ($\pm 15\%$).

The estimates of the data-driven fake-lepton background also have uncertainties specific to each channel. In the $\ell \nu \ell \nu \ell \nu$ channel, the systematic uncertainty results from the uncertainties on the probabilities of candidate leptons that satisfy the looser lepton selection criteria to also satisfy the signal lepton selection criteria. For prompt leptons this uncertainty is ±(5 to 10)% while for fake leptons and misidentified/non-prompt leptons this uncertainty is ±(80 to 90)%. The latter uncertainty is a conservative estimate which accounts for differences in the heavy-flavour and light-flavour composition between the signal region and the control region where the fake-lepton efficiency is determined for these leptons. In the $\ell \nu \ell \nu jj$ channel, the systematic uncertainty results from the uncertainties in the measurement of the fake factors, which is estimated to be ±(20 to 30)%. Statistical uncertainties in the samples used for the matrix method and the fake-factor method also contribute to the overall uncertainty of the estimation of the fake-lepton background. The total uncertainty in the overall fake-lepton background yield is $\pm 13\%$ in the $\ell \nu \ell \nu \ell \nu$ channel and $\pm 8\%$ in the $\ell \nu \ell \nu jj$ channel.

The charge-flip background is only relevant for the $e^\pm e^\pm$/$e^\pm \mu ^\pm$ final state in the $\ell \nu \ell \nu jj$ channel and for the 0-SFOS region in the $\ell \nu \ell \nu \ell \nu$ channel. Its uncertainty is dominated by the statistical precision with which the electron charge misidentification rate is determined from the available data. Since the charge-flip background estimation uses the number of $Z(\rightarrow e^+ e^-)+2$ jets events, the number of events in the data also contributes to the overall systematic uncertainty. In the $\ell \nu \ell \nu \ell \nu$ channel, the uncertainty on the charge-flip background estimate is $\pm 0.5\%$ in the 0-SFOS region but is $\pm 0.04\%$ for the total background estimate in all three signal regions. In the $\ell \nu \ell \nu jj$ channel, the total systematic uncertainty in the overall background yield due to the uncertainty in the charge-flip background estimate is found to be $\pm 2.2\%$.

There are also uncertainties in the overall normalization of the signal and MC background cross sections. Uncertainties in the signal cross section are those described in Sect. 4. These are not, however, included as uncertainties in the model and merely serve as a comparison for the final measurement in Sect. 7. The normalizations of the SM background cross sections described in Sect. 5.1 have their own associated uncertainties. The uncertainty in the predicted *WZ*+jets background cross section is the most important one since it is the largest irreducible background. The size of the uncertainty relative to the predicted *WZ*+jets background is $\pm 10\%$ in the $\ell \nu \ell \nu \ell \nu$ channel and ±(16 to 23)% in the $\ell \nu \ell \nu jj$ channel depending on the production mechanism. This uncertainty is based on the measurement performed in the control region, for the $\ell \nu \ell \nu \ell \nu$ channel as described in Sect. 5, while it is a combination of the scale, PDF and parton shower uncertainties estimated as in Ref. [12], for the $\ell \nu \ell \nu jj$ channel. The remaining uncertainties are mostly negligible in the overall background prediction. The normalization uncertainty in the total background prediction is around $\pm 8$% in the $\ell \nu \ell \nu \ell \nu$ channel and $\pm 13$% in the $\ell \nu \ell \nu jj$ channel.

The uncertainty on the integrated luminosity is $\pm 1.9\%$, affecting the overall normalization of both the signal and background processes estimated from MC simulation. It is derived following the methodology detailed in Ref. [18]. The uncertainties associated with the pile-up reweighting of the events are estimated to be no more than $\pm 0.1\%$ for the signal and the backgrounds.

## Cross-section measurement

**Fig. 3:**
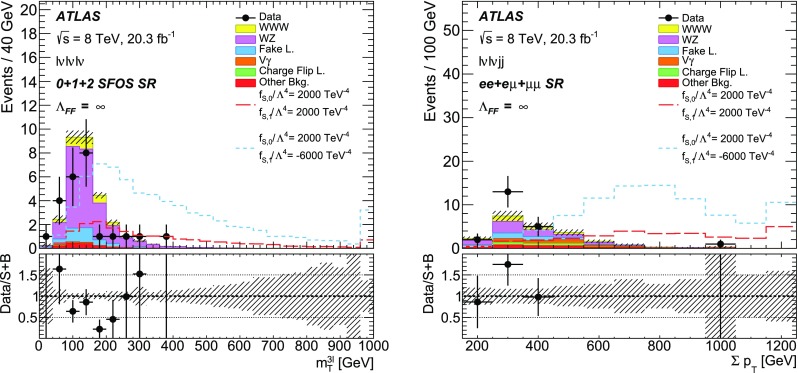
The distribution of $m_T^{3\ell }$ for the $\ell \nu \ell \nu \ell \nu$ channel (*left*) and the distribution of $\Sigma p_{\text {T}}$ for the $\ell \nu \ell \nu jj$ channel (*right*) as observed in the data (*dots with error bars* indicating the statistical uncertainties) and as expected from SM signal and background processes. The ratios between the observed numbers of events in data and the expected SM signal plus background contributions are shown in the lower panels. The *hashed bands* results from the systematic uncertainties on the sum of the signal plus background contributions. The “other backgrounds” contain prompt leptons and are estimated from MC. Contributions from aQGCs are also shown, assuming the non-unitarized case ($\Lambda _\mathrm{FF} = \infty$) and two different sets of $f_{S,0}/\Lambda ^4$ and $f_{S,1}/\Lambda ^4$ configurations ($f_{S,0}/\Lambda ^4{}=2000~\text {TeV}{}^{-4}$, $f_{S,1}/\Lambda ^4{}=2000~\text {TeV}{}^{-4}$ and $f_{S,0}/\Lambda ^4{}=2000~\text {TeV}{}^{-4}$, $f_{S,1}/\Lambda ^4{}=-6000~\text {TeV}{}^{-4}$). The highest bin also includes events falling out of the range shown

The signal and background predictions together with their uncertainties are compared to the data for six signal regions in Table 5. The expected signal yields are calculated using the SM $W^{\pm }W^{\pm }W^{\mp }$ cross sections listed in Sect. 4. The expected numbers of signal plus background events are consistent with the numbers of events observed in data in all regions. Figure 3 shows the $m_{T}^{3\ell }$ distribution for the $\ell \nu \ell \nu \ell \nu$ channel and the distribution of the sum of the scalar $p_{\text {T}}$ for all selected objects, $\Sigma p_{\text {T}} = p_{\text {T}} ^{\ell ,1}+p_{\text {T}} ^{\ell ,2}+p_{\text {T}} ^{j,1}+p_{\text {T}} ^{j,2}+E_{\text {T}}^{\text {miss}}$, for the $\ell \nu \ell \nu jj$ channel, after summing over the three signal regions in each channel. Good agreement between data and the signal-plus-background model is observed for both distributions.

**Table 5:** Numbers of expected signal and background events, and their statistical and systematic uncertainties, together with the observed yields in the data in the signal regions for the two channels

$\ell \nu \ell \nu \ell \nu$	0 SFOS					1 SFOS					2 SFOS				
$W^{\pm }W^{\pm }W^{\mp }$ signal	1.34	±	0.02	±	0.07	1.39	±	0.02	±	0.08	0.61	±	0.01	±	0.03
*WZ*	0.59	±	0.00	±	0.07	11.9	±	0.1	±	1.3	9.1	±	0.1	±	1.0
Other prompt background	0.21	±	0.01	±	0.02	0.78	±	0.02	±	0.11	0.60	±	0.02	±	0.10
Charge-flip background	0.04	±	0.00	±	0.01	–					–				
$V\gamma$	–					0.20	±	0.13	±	0.29	0.11	±	0.10	±	0.29
Fake-lepton background	1.5	±	0.3	±	1.4	1.9	±	0.3	±	1.9	0.49	±	0.16	±	0.47
Total background	2.4	±	0.3	±	1.4	14.8	±	0.4	±	2.3	10.3	±	0.2	±	1.2
Signal $+$ background	3.7	±	0.3	±	1.4	16.2	±	0.4	±	2.3	10.9	±	0.2	±	1.2
Data	5					13					6				

$\ell \nu \ell \nu jj$	$e^\pm e^\pm$					$e^\pm \mu ^\pm$					$\mu ^\pm \mu ^\pm$				
$W^{\pm }W^{\pm }W^{\mp }$ signal	0.46	±	0.03	±	0.07	1.35	±	0.05	±	0.19	1.65	±	0.06	±	0.30
*WZ*	0.74	±	0.13	±	0.44	2.77	±	0.27	±	0.66	3.28	±	0.29	±	0.71
Other prompt background	0.46	±	0.05	±	0.16	1.33	±	0.10	±	0.38	1.33	±	0.15	±	0.38
Charge-flip background	1.13	±	0.13	±	0.24	0.74	±	0.08	±	0.16	–				
$V\gamma$	0.75	±	0.35	±	0.21	2.5	±	0.7	±	0.7	–				
Fake-lepton background	0.96	±	0.15	±	0.39	2.04	±	0.22	±	0.89	0.43	±	0.06	±	0.25
Total background	4.0	±	0.4	±	0.7	9.4	±	0.8	±	1.4	5.0	±	0.3	±	0.8
Signal $+$ background	4.5	±	0.4	±	0.7	10.7	±	0.8	±	1.4	6.7	±	0.3	±	0.9
Data	0					15					6				

The amount of $W^{\pm }W^{\pm }W^{\mp }$ signal in the selected data set is determined using the numbers of expected signal and background events as well the numbers of observed events in the data. The signal strength, $\mu$, is the parameter of interest, defined as a scale factor multiplying the cross section times branching ratio predicted by the SM. A test statistic based on the profile-likelihood ratio [58] is used to extract $\mu$ from a maximum-likelihood fit of the signal-plus-background model to the data. The likelihood, $\mathcal {L}$, is given by

1 $$\begin{aligned} \mathcal{{L}} = \prod _c \prod _i {\text {Poisson}} \left[ n_{i_c}^{\text {obs}} \mid \mu \times n_{i_c}^{\text {sig, SM}} (\theta _k) + n_{i_c}^{\text {bkg}}(\theta _k) \right] \prod _k g(\theta _k) \end{aligned}$$

where the index *c* represents one of the two analysis channels, *i* represents one of the three signal regions in each channel, $n^{\text {obs}}$ is number of observed events, $n^{\text {sig, SM}}$ is the expected number of signal events based on the SM calculations, and $n^{\text {bkg}}$ is the expected number of background events. The effect of a systematic uncertainty *k* on the likelihood is modelled with a nuisance parameter, $\theta _k$, constrained with a corresponding Gaussian probability density function $g(\theta _k)$.

The test statistic, $t_\mu$, is defined as

2 $$\begin{aligned} t_{\mu } = -2 \ln \lambda (\mu )=-2 \ln \frac{\mathcal{{L}}(\mu , \hat{\hat{\theta }}(\mu ))}{\mathcal{{L}}(\hat{\mu }, \hat{\theta })} \end{aligned}$$

where $\hat{\mu }$ is the unconditional maximum-likelihood (ML) estimators of the independent signal strength in the categories being compared, $\hat{\theta }$ are the unconditional ML estimators of the nuisance parameters, and $\hat{\hat{\theta }}(\mu )$ are the conditional ML estimators of $\theta$ for a given value of $\mu$. The significance of $\mu$ is obtained with the above test statistic, and is estimated using 100,000 MC pseudo-experiments to determine how well the fit result agrees with the background-only hypothesis. The observed (expected) significance of a positive signal cross section is $0.96~\sigma$ ($1.05~\sigma$) for the combination of the two channels. Most of the sensitivity comes from the 0-SFOS category in the $\ell \nu \ell \nu \ell \nu$ channel and the $\mu ^\pm \mu ^\pm$ category in the $\ell \nu \ell \nu jj$ channel. The most significant deviation from the signal-plus-background hypothesis occurs in the $e^\pm e^\pm$ region, where zero events are observed and 4.0 background and 0.46 signal events are expected. The probability that the background fluctuates down to zero events is 2.3%.

**Fig. 4:**
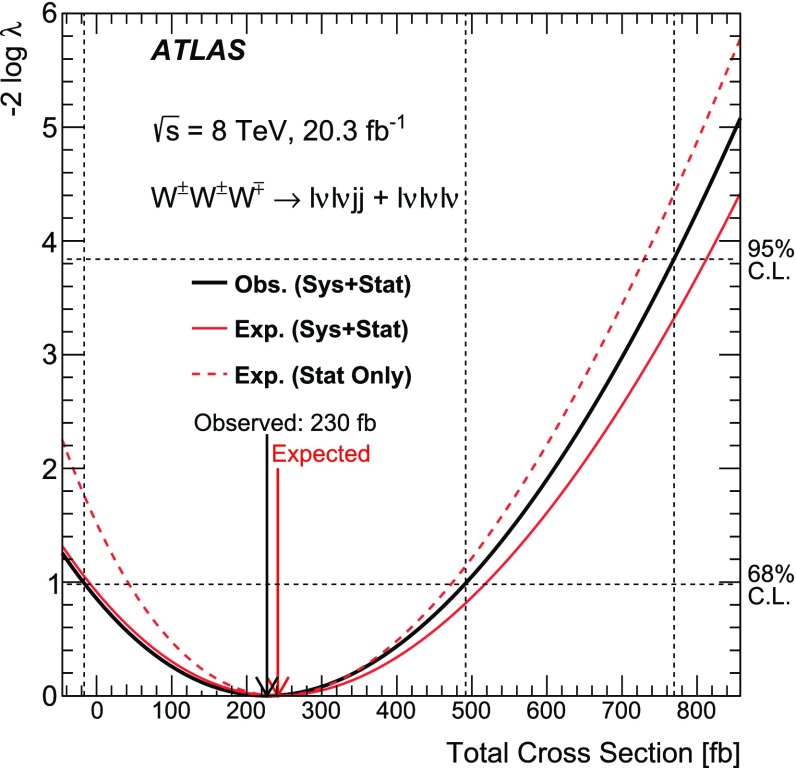
Profile-likelihood scans as a function of the total cross section for the combination of all six signal regions. The expected (*red*) scans are shown when considering only statistical uncertainties (*dashed line*) and when considering both statistical and systematic uncertainties (*solid line*). The observed (*black solid line*) scan is also shown. The *dotted black grid-lines* pinpoint the location of the 68 and 95% CL uncertainties in the measurement of the signal strength

The central value of $\mu$ corresponds to the minimum of the negative log-likelihood distribution. The measured fiducial cross section in each channel is obtained by multiplying $\mu$ by the expected value of the fiducial cross section in that channel. The measured total cross section is obtained by combining the results for the two channels and then extrapolating to the total phase space using the signal acceptance. The log-likelihood scans for the total cross-section measurement are evaluated with and without systematic uncertainties and are shown in Fig. 4. The expected and observed fiducial and total cross sections are summarized in Table 6.

The presence of the $W^{\pm }W^{\pm }W^{\mp }$ signal is also assessed using a one-sided 95% CL upper limit on the production cross section using the CL$_s$ method of Ref. [59]. The limits are evaluated using 2000 MC pseudo-experiments. The observed (expected) upper limit on the fiducial cross section in the absence of $W^{\pm }W^{\pm }W^{\mp }$ production is found to be 1.3 fb (1.1 fb) in the $\ell \nu \ell \nu \ell \nu$ channel and 1.1 fb (0.9 fb) in the $\ell \nu \ell \nu jj$ channel. The observed (expected) upper limit in the absence of $W^{\pm }W^{\pm }W^{\mp }$ production on the total cross section is 730 fb (560 fb) when the two channels are combined. If the SM $W^{\pm }W^{\pm }W^{\mp }$ signal is also considered, the expected upper limit on the total cross section is 850 fb.

**Table 6:** The predicted and observed fiducial cross sections for the $\ell \nu \ell \nu \ell \nu$ and $\ell \nu \ell \nu jj$ channels and the predicted and observed total cross section for the combination of the two channels

	Cross section (fb)	
	Theory	Observed
Fiducial		
$\ell \nu \ell \nu \ell \nu$	$0.309 \pm 0.007~(\mathrm{stat.})~\pm 0.015~(\mathrm{PDF}) \pm 0.008~(\mathrm{scale})$	$0.31~^{+0.35}_{-0.33}$ (stat.) $^{+0.32}_{-0.35}$ (syst.)
$\ell \nu \ell \nu jj$	$0.286 \pm 0.006~(\mathrm{stat.})~\pm 0.015~(\mathrm{PDF}) \pm 0.010~(\mathrm{scale})$	$0.24~^{+0.39}_{-0.33}$ (stat.) $^{+0.19}_{-0.19}$ (syst.)
Total	$241.5 \pm 0.1$ (stat.) $\pm ~10.3$ (PDF) $\pm ~6.3$ (scale)	$230~{\pm 200}$ (stat.) $^{+150}_{-160}$ (syst.)

## Limits on anomalous quartic gauge couplings (aQGCs)

Contributions from sources beyond the SM to the $W^{\pm }W^{\pm }W^{\mp }$ production process can be expressed in a model-independent way using higher-dimensional operators leading to *WWWW* aQGCs. The parameterization of aQGCs is based on Ref. [60] in a linear representation [61] considering only dimension-eight operators involving four gauge bosons. There are 18 dimension-eight operators built from the covariant derivative of the Higgs field $D_\mu \Phi$, the SU(2)$_{\text {L}}$ field strength $W^i_{\mu \nu }$, and U(1)$_{\text {Y}}$ field strength $B_{\mu \nu }$. Only the two terms built exclusively from $D_\mu \Phi$ and with aQGC parameters $f_{S,0}/\Lambda ^4$ and $f_{S,1}/\Lambda ^4$ are considered in this analysis:

3 $$\begin{aligned} \mathcal{{L}}_{S,0} = \frac{f_{S,0}}{\Lambda ^4} [(D_\mu \Phi )^\dagger D_\nu \Phi ] \times [(D^\mu \Phi )^\dagger D^\nu \Phi ], \end{aligned}$$

4 $$\begin{aligned} \mathcal{{L}}_{S,1} = \frac{f_{S,1}}{\Lambda ^4} [(D_\mu \Phi )^\dagger D^\mu \Phi ] \times [(D_\nu \Phi )^\dagger D^\nu \Phi ], \end{aligned}$$

where $\Lambda$ is the energy scale of the new physics. These two operators only affect massive bosons and do not depend on the gauge boson momenta since no SU(2)$_{\text {L}}$ or U(1)$_{\text {Y}}$ field strengths are included. As a result, they are important for the study of longitudinal vector-boson scattering. Similar parameters were studied before by the ATLAS and CMS Collaborations in Refs. [8, 10, 16]

The effective Lagrangian approach leads to tree-level unitarity violation. This can be avoided by introducing a form factor [62] as

5 $$\begin{aligned} \alpha \rightarrow \frac{\alpha _0}{(1+\hat{s}/\Lambda _\mathrm{FF}^2)} \end{aligned}$$

where $\alpha$ corresponds to one of the two couplings, $\alpha _0$ is the value of the aQGC at low energy, $\hat{s}$ is the square of the partonic centre-of-mass energy, and $\Lambda _\mathrm{FF}$ is the form-factor cutoff scale. However, there is no theoretical algorithm to predict for which form-factor cutoff scale the cross section would violate unitarity. Therefore different values of $\Lambda _\mathrm{FF}$ are considered with $\Lambda _\mathrm{FF}=$ 0.5, 1, 2, and 3 $\text {TeV}$ as well as $\Lambda _\mathrm{FF} = \infty$, which corresponds to the non-unitarized case.

**Fig. 5:**
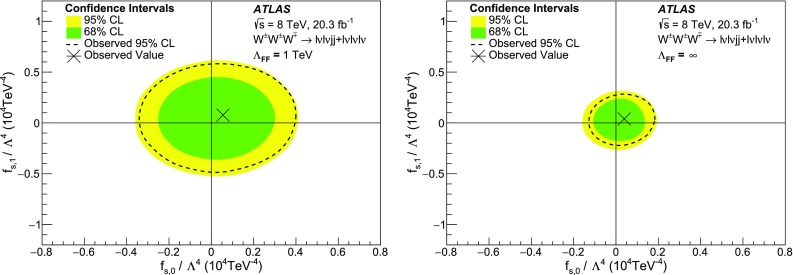
Expected 68 and 95% CL contours for $f_{S,1}/\Lambda ^4$ vs $f_{S,0}/\Lambda ^4$ compared to the observed 95% CL contour and the observed best-fit value for cases when $\Lambda _\mathrm{FF}=1$ $\text {TeV}$ (*left*) and $\Lambda _\mathrm{FF}=\infty$ (*right*)

Events with aQGCs are generated with vbfnlo at LO and passed through the ATLAS detector simulation. A grid of samples is obtained using different parameters of $f_{S,0}/\Lambda ^4{}$ and $f_{S,1}/\Lambda ^4{}$ values. The interpolation between these points is performed with a 2-dimensional quadratic function in the ($f_{S,0}/\Lambda ^4{}$, $f_{S,1}/\Lambda ^4{}$) space. The LO samples are scaled using a factor derived from the ratio of the SM LO to NLO predictions. Figure 3 show the expected distribution for the non-unitarized ($\Lambda _\mathrm{FF} = \infty$) aQGC signal samples being generated with parameters $f_{S,0}/\Lambda ^4{}=2000~\text {TeV}{}^{-4}$, $f_{S,1}/\Lambda ^4{}=2000~\text {TeV}{}^{-4}$ in red and parameters $f_{S,0}/\Lambda ^4{}=2000~\text {TeV}{}^{-4}$, $f_{S,1}/\Lambda ^4{}=-6000~\text {TeV}{}^{-4}$ in blue as a function of the $m_T^{3\ell }$ distribution in the $\ell \nu \ell \nu \ell \nu$ channel and the $\Sigma p_{\text {T}}$ distribution in the $\ell \nu \ell \nu jj$ channel, summed over the three signal regions in each channel. Even though aQGC events tend to have leptons or jets with larger momenta, the detection efficiency for events in the fiducial region is found to be consistent with the one obtained for the SM sample within 20%. The efficiencies of the aQGC samples are used with their statistical and systematic uncertainties to derive the 95% confidence intervals (CI) on aQGC, while the largest observed deviation of the aQGC efficiencies from the SM one is used as an extra systematic uncertainty. Frequentist CI on the anomalous coupling are computed by forming a profile-likelihood-ratio test that incorporates the observed and expected numbers of signal events for different values of the anomalous couplings. Table 7 shows the expected and observed 95% CI on $f_{S,0}/\Lambda ^4$ ($f_{S,1}/\Lambda ^4$) with different $\Lambda _\mathrm{FF}$ values, assuming $f_{S,1}/\Lambda ^4$ ($f_{S,0}/\Lambda ^4$) to be zero. Figure 5 shows the two-dimensional 95% CL contour limits of $f_{S,0}/\Lambda ^4$ vs $f_{S,1}/\Lambda ^4$ in the cases where $\Lambda _\mathrm{FF} = 1$ $\text {TeV}$ and $\Lambda _\mathrm{FF} = \infty$. For $\Lambda _\mathrm{FF} = \infty$, the limits can be compared to the stronger limits obtained by the CMS Collaboration in Ref. [16] in a different production channel. Other parameterization ($\alpha _4$, $\alpha _5$) of new physics have been introduced in Refs. [63–65]. The limits presented in this paper can be converted into limits on $\alpha _4$ and $\alpha _5$ following the formalism defined in the Appendix of Ref. [60] and using Equations (60) and (61) in Ref. [66]. For example, non-unitarized limits obtained for $\Lambda _\mathrm{FF} = \infty$ are: $\alpha _4$ expected [$-0.61$, 0.78], $\alpha _4$ observed [$-0.49$, 0.75] and $\alpha _5$ expected [$-0.57$,0.69], $\alpha _5$ observed [$-0.48$,0.62]. Limits derived by the ATLAS Collaboration in other final states are reported in Refs. [8, 10]. The latter were obtained using a different unitarization scheme. Since that scheme is not applicable to triboson production, a combination of the limits is not possible.

**Table 7:** Expected and observed 95% CI on $f_{S,0}/\Lambda ^4$ ($f_{S,1}/\Lambda ^4$) for different $\Lambda _\mathrm{FF}$ values, assuming $f_{S,1}/\Lambda ^4$ ($f_{S,0}/\Lambda ^4$) to be zero

$\Lambda _\mathrm{FF}$ ($\text {TeV}$)	Expected CI ($\times 10^4\,$ $\text {TeV}$ $^{-4}$)		Observed CI ($\times 10^4\,$ $\text {TeV}$ $^{-4}$)	
	$f_{S,0}/\Lambda ^4$	$f_{S,1}/\Lambda ^4$	$f_{S,0}/\Lambda ^4$	$f_{S,1}/\Lambda ^4$
0.5	[$-0.79$, 0.89]	[$-1.06$, 1.27]	[$-0.74$, 0.86]	[$-0.99$, 1.20]
1	[$-0.36$, 0.41]	[$-0.52$, 0.60]	[$-0.34$, 0.40]	[$-0.48$, 0.58]
2	[$-0.22$, 0.25]	[$-0.33$, 0.39]	[$-0.20$, 0.24]	[$-0.29$, 0.36]
3	[$-0.19$, 0.22]	[$-0.29$, 0.36]	[$-0.16$, 0.21]	[$-0.25$, 0.33]
$\infty$	[$-0.16$, 0.19]	[$-0.25$, 0.30]	[$-0.13$, 0.18]	[$-0.21$, 0.27]

## Summary

A search for triboson $W^{\pm }W^{\pm }W^{\mp }$ production in two decay channels (${W^{\pm }W^{\pm }W^{\mp } \rightarrow \ell ^\pm \nu \ell ^\pm \nu \ell ^\mp \nu }$ and ${W^{\pm }W^{\pm }W^{\mp } \rightarrow \ell ^\pm \nu \ell ^\pm \nu jj}$ with $\ell =e, \mu$) is reported, using proton-proton collision data corresponding to an integrated luminosity of 20.3 $\mathrm{fb}^\mathrm{-1}$ at a centre-of-mass energy of 8 $\text {TeV}$ collected by the ATLAS detector at the LHC. Events with exactly three charged leptons or two same-charge leptons in association with two jets are selected. The data are found to be in good agreement with the SM predictions in all signal regions. The observed 95% CL upper limit on the SM $W^{\pm }W^{\pm }W^{\mp }$ production cross section is found to be 730 fb with an expected limit of 560 fb in the absence of $W^{\pm }W^{\pm }W^{\mp }$ production. Limits are also set on the aQGC parameters $f_{S,0}/\Lambda ^4$ and $f_{S,1}/\Lambda ^4$.

## Footnotes

1 The ATLAS experiment uses a right-handed coordinate system with its origin at the nominal interaction point (IP) in the centre of the detector. The *x*-axis points from the IP to the centre of the LHC ring, the *y*-axis points upward, and the *z*-axis is along one of the proton beam directions. Cylindrical coordinates $(r,\phi )$ are used in the transverse plane, $\phi$ being the azimuthal angle around the beam pipe. The pseudorapidity is defined in terms of the polar angle $\theta$ as $\eta =-\ln \tan (\theta /2)$. Transverse momentum ($p_{\text {T}}$) is defined relative to the beam axis and is calculated as $p_{\text {T}} =p \sin \theta$ where *p* is the momentum.
